# GDEE: A Structure-Based Platform for Gene Discovery and Enzyme Engineering

**DOI:** 10.1021/acs.jcim.5c02688

**Published:** 2026-09-22

**Authors:** Caio S. Souza, João P. G. Correia, Isabel Rocha, Diana Lousa, Cláudio M. Soares

**Affiliations:** Instituto de Tecnologia Química e Biológica António Xavier, 98819 Universidade Nova de Lisboa, Av. da República, 2780-157 Oeiras, Portugal

## Abstract

Enzymes are extensively utilized for catalyzing the production of value-added compounds in diverse sectors with significant economic impact. However, challenges such as poor protein expression, low catalytic activity, substrate/co-factor limitations, and toxicity of final products limit the efficiency of these biocatalysts. Protein engineering offers a solution by redesigning enzyme catalytic properties to enhance biosynthetic pathways. In this study, we present an automated platform for gene discovery and enzyme engineering, aimed at overcoming these bottlenecks. The GDEE platform finds and optimizes enzymes responsible for rate-limiting steps in biosynthetic pathways, with objectives ranging from improving catalytic efficiency to enabling novel transformations. The platform operates through four key steps: an initial sequence step that either sources natural enzyme sequences or generates mutant variants, followed by an atomistic protein structure prediction step, a docking step, and a binding energy evaluator, obtaining a set of variant enzyme sequences that are prioritized for experimental validation. Additionally, machine learning-based re-scoring of binding poses to improve binding affinities significantly enhances the accuracy of predicting the effect of mutations on binding, highlighting its potential in metabolic engineering applications.

## Graphical abstract

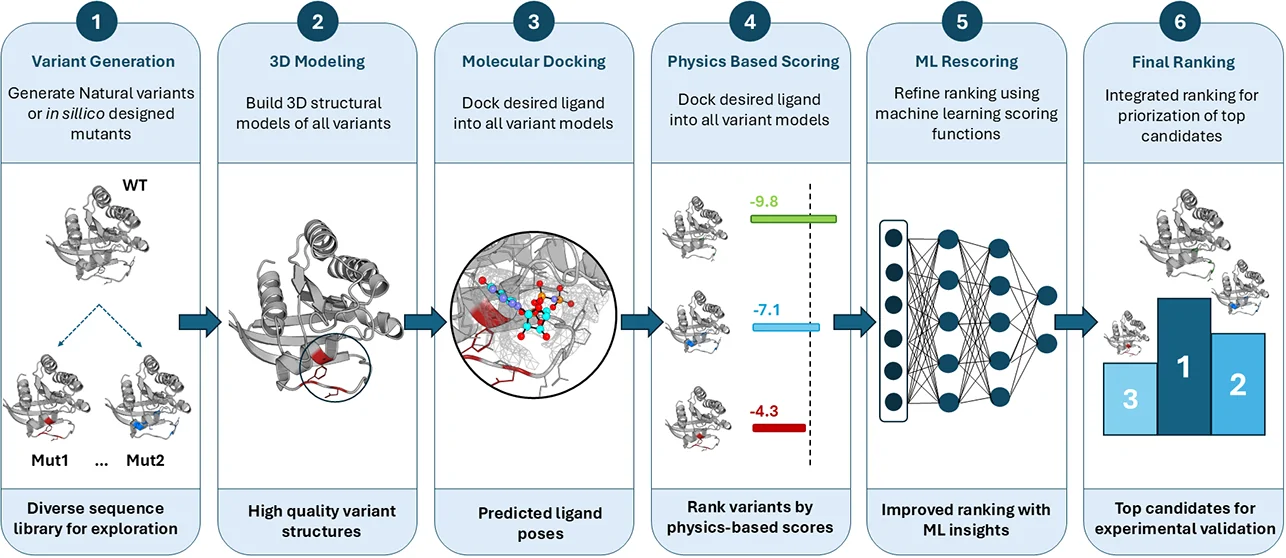

## Introduction

Poor enzyme expression, low catalytic levels, low cofactor or substrate concentrations, toxicity toward the end product, and other variables can all contribute to low performance of natural or novel pathways in microorganisms.^1,2^ Improving enzymatic DNA transcription has been the most popular technique for overcoming some of these challenges. However, other difficulties such as cofactor availability, low substrate affinity, or inhibition of catalysis, cannot be addressed by increasing enzyme concentration. Enzyme engineering to optimize metabolic pathways is an effective strategy for re-designing the catalytic characteristics to favor a certain flow.^3^ It is feasible to enhance the catalytic properties by increasing substrate affinity and selectivity, or to eliminate inhibition.^4^

Enzyme performance can be improved through different mechanisms. Catalytic efficiency is commonly described by

$$\frac{k_{cat}}{K_{M}}$$

and can be increased by enhancing the turnover number, *k*_cat_, by decreasing the apparent Michaelis constant, *K*_M_, or by affecting both terms. Mutations that improve substrate recognition, active-site complementarity, or productive ligand orientation may contribute to lower apparent *K*_M_ values or altered substrate selectivity. However, improved substrate binding does not necessarily imply improved catalytic efficiency, because turnover also depends on transition-state stabilization and other factors. Therefore, binding-based predictions should be interpreted as a means to prioritize variants for experimental testing, rather than as direct predictions of catalytic improvement.

Several techniques and protocols have been created, in order to do enzyme engineering, employing random mutagenesis and selection via direct evolution, or hybrid methods where rational design is employed to reduce the reliance on widespread random mutagenesis.^5^

Computational tools are also important in enzyme engineering. When the structure of the enzyme is available, rational design approaches may be used to predict how amino acid substitutions affect protein structure, stability, ligand binding, and, indirectly, enzymatic performance. Several structure-based methods have been developed for this purpose. For example, Rosetta^6^ has been widely used for protein design, mutation modeling, and enzyme engineering through conformational sampling and energy-based scoring. FoldX^7^ estimates the energetic effects of mutations on protein stability and protein interactions using an empirical force field, while DynaMut^8^ predicts mutation-induced changes in protein stability by integrating normal mode analysis with graph-based signatures of protein dynamics. These tools illustrate the importance of structure-based modeling in protein engineering and provide complementary strategies for evaluating the consequences of amino acid substitutions. However, their direct application to large-scale enzyme variant prioritization may require additional automation, integration with ligand docking, and workflow standardization.

In rational protein engineering, several modeling methods can be utilized, ranging from highly detailed low-throughput methods to approaches that enable higher-throughput analysis by introducing approximations. In the first group, Quantum Mechanics calculations and combined Quantum Mechanics/Molecular Mechanics techniques can be used. These approaches are particularly detailed because they treat the enzyme active site at the quantum level and allow analysis of reaction mechanisms, as well as prediction of activation barriers. However, they are generally too computationally demanding for high-throughput calculations.^9–11^

Molecular mechanics (MM) based free energy calculations and other MM-based methods, such as meta dynamics, can also be used to calculate free energy differences and thus predict how mutations affect the binding of a given substrate or reaction intermediate, but these methods are also computationally demanding.^10^

Molecular docking approaches are much faster due to the numerous approximations introduced (such as assuming the protein rigid or semi-rigid and not employing explicit solvent) and predict the binding free energy of a ligand to a protein by applying empirical free energy functions.^12^ Although docking does not explicitly model the chemical reaction step or directly predict *k*_cat_, it can be valuable for enzyme engineering problems where substrate binding is expected to be a limiting step, being able to decrease the apparent *K*_M_ constant. Docking techniques have been used successfully in rational enzyme design,^13^ and, although not as thorough as QM-based approaches, these methods are faster and may be employed in a high-throughput manner. AutoDock Vina is especially well-suited for this task, in view of its calculation efficiency, allowing it to run faster without sacrificing prediction of binding poses and energies.^14^ To apply docking approaches in a high-throughput manner, one must have an automated way of generating and preparing mutant enzyme structures as well as executing docking calculations.

To overcome this problem, we developed an automated platform that combines several structural bioinformatic tools that can be used to perform structure-based (i) enzyme engineering by mutating specific residues of a given active site to optimize the enzyme transformation, or (ii) gene discovery, where natural enzymes are scanned to find those that are more prone to catalyze the target reaction. This platform will predict which of the candidate enzymes has a better affinity for the molecule of interest (substrate, intermediate or product) given an initial set of enzymes (which can be *in-silico* generated mutants of the same enzyme or natural occurring variants). Rather than calculating catalytic efficiency directly, which is computationally demanding and often unfeasible for large variant libraries due to the complexity of modeling full enzymatic reaction mechanisms, this platform uses predicted binding affinity as a practical proxy. Binding affinity indicates how strongly an enzyme can bind to its target molecule. and can often be evaluated computationally for large numbers of candidates.^15–17^ In enzyme engineering, ligand binding is frequently an important component of catalytic performance, particularly when substrate recognition, intermediate stabilization, product release, cofactor binding, or inhibitor interactions influence activity or selectivity. However, binding affinity alone does not fully determine catalytic efficiency, which also depends on several factors.

Accordingly, GDEE is designed as a computational prioritization platform rather than a definitive predictor of catalytic improvement. By estimating how mutations may affect ligand binding and binding pose, the platform can help selecting promising candidate variants before experimental testing, thereby supporting medium- to high-throughput enzyme engineering workflows.

## Overview

The gene discovery and enzyme engineering pipeline consist of a sequence of steps aiming at designing putatively more efficient variants of enzymes. The Pipeline steps are grouped into four main steps: an initial sequence step that either sources natural enzyme sequences or generates mutant variants, followed by an atomistic protein structure prediction step, a docking step, and a binding energy evaluator. The platform, at this stage, assumes that a protein with a set of mutations that increases the enzyme affinity to substrates, products or reaction intermediates is prone to have better kinetic parameters. Thus, with this pipeline, it is possible to generate a set of variant enzymes and filter which sequences are putative candidates for experimental expression and enzymatic testing.

## GDEE Platform

The GDEE platform has been developed for some years and applied to optimize several transformations (some already published^18,19^ ) but never published in detail. Here we are presenting a consolidated version, which is usable by the scientific community. GDEE is developed as a Python package composed of 8 distinct modules, as illustrated in Figure 1. Each module serves a specific role within the enzyme engineering or gene discovery pipeline. The Pipeline module orchestrates the pipeline workflow and data persistence. The **Variant** module gathers protein sequence variants using different strategies, including natural or mutant sequences. The **Modeling** module predicts the three-dimensional structure of each variant and assesses the quality of the modeled structures. The **Evaluator** module performs protein-ligand docking and calculates binding affinities. The **Measurement** module computes distances between selected atoms of the protein and ligand to support filtering of docking poses. The **Analysis** module filters docking poses based on user defined metrics and ranks the enzyme variants accordingly. The **Platform** module manages execution modes, supporting both single-threaded and distributed workflows for scalability. Finally, the **Database** module handles all data persistence, utilizing SQLite3 to store and organize data for effective post-processing and re-analysis.

**Figure 1:**
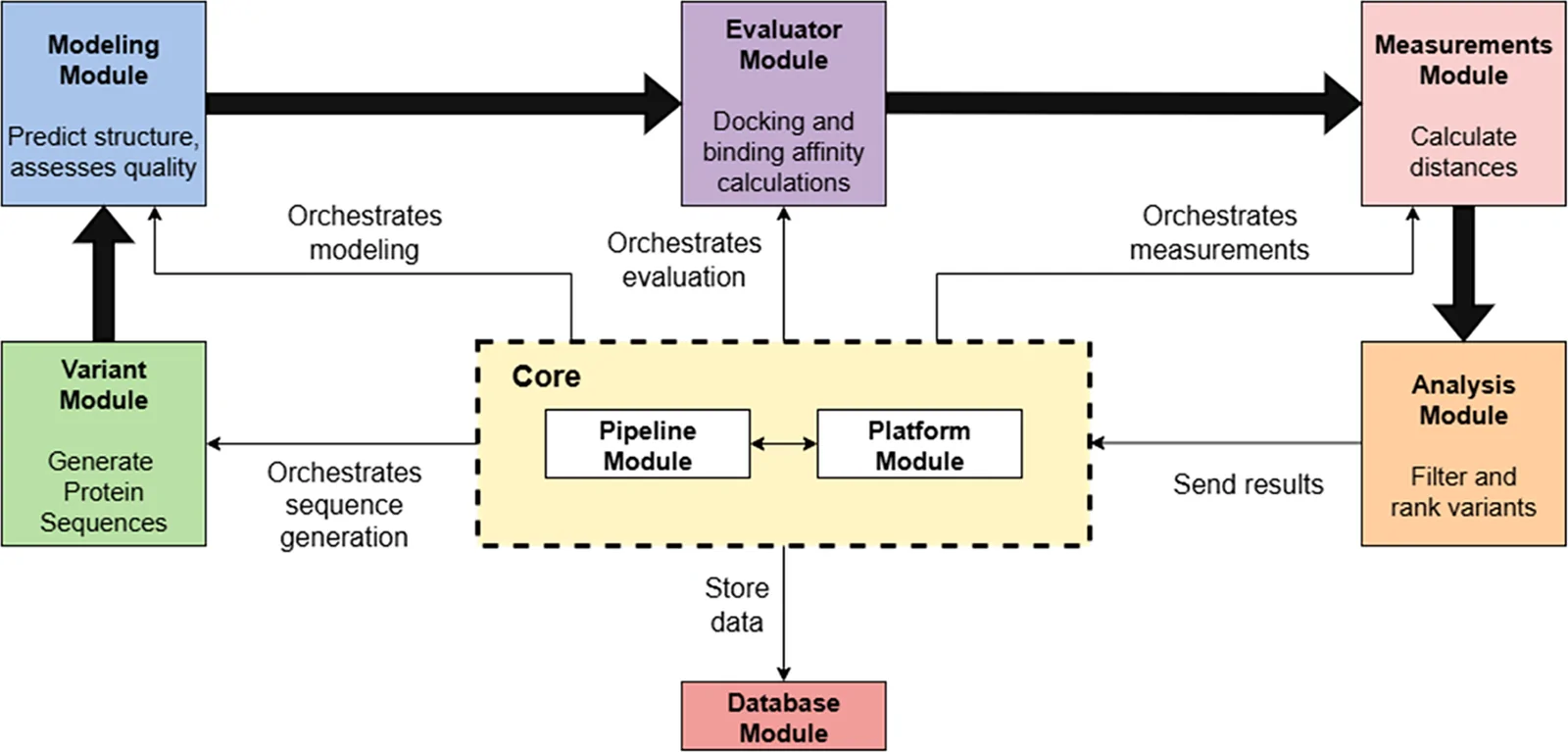
High-level diagram illustrating the architecture and data flow of the platform. The **Core** functions as the central orchestrator coordinating and scheduling task execution across specialized modules in a linear pipeline workflow—**Variant**, **Modeling**, **Evaluator**, **Measurement**, and **Analysis**—each dedicated to specific tasks on protein variants. The **Variant** module generates amino acid sequence variants using different strategies; the **Modeling** module predicts 3D structures and assesses their quality; the **Evaluator** module performs docking and binding affinity calculations; the **Measurement** module calculates distances between selected protein and ligand atoms to assist in filtering; and the **Analysis** module applies filters and ranks variants accordingly. Data flows between processing modules are represented by bold arrows, while narrow arrows indicate the **Core** orchestration and management of operations. All intermediate and final results are centralized in the **Database** module, providing efficient data storage and retrieval for downstream analysis.

The package is designed with an object-oriented and modular architecture that facilitates extensibility. New algorithms and functionalities can be seamlessly integrated, allowing customization and growth of the platform.

External software dependencies play a key role in the pipeline. The modeling module uses Modeller^20^ via its Python package integration for structure prediction. Docking and affinity calculations rely on AutoDock Vina^14^ or Smina.^21^ Parallel processing is implemented using MPI4Py,^22^ a Python interface to the Message Passing Interface (MPI), enabling efficient data parallelism across computational resources.

All results and intermediate data are stored in a SQLite3 database, ensuring organized data management and facilitating downstream analysis and reproducibility.

## Gene Discovery and Enzyme Engineering Pipeline

The initial input for each job is the structure of the template enzyme and the structure of one or more ligand molecules. The molecular structures of the protein and ligand are expected to be in a PDB (Protein Data Bank) and PDBQT file format, respectively. After providing the initial data it is possible to create a new job that will proceed with the pipeline steps. The pipeline starts with the generation of a set of candidate enzyme sequences to be evaluated. Multiple homology models are built and optimized for each sequence variant using the user-provided template structure. The final models undergo molecular docking calculations with a ligand that represents the reaction’s limiting step (usually the reaction intermediate or substrate). Finally, the sequences are ranked according to the binding affinity computed in the docking step.

### Sequence Generation Step

The gene discovery and the enzyme engineering features differ only in the sequence generation step. For enzyme engineering, sequences are automatically created by sampling the mutation space of user-selected residues or by generating all possible combinations of amino acids for each residue position selected. In the case of sampling, a user-defined number of amino acid residue substitutions is chosen based on the scoring of a substitution matrix for each individual annotated site. Mutations may be conservative (lowest substitution cost), non-conservative (highest substitution cost) or completely random (substitution matrix is ignored). The platform can generate new sequences by applying groups of mutations of varying sizes, allowing simultaneous substitutions of one, two, three, or more amino acids according to the desired mutations count. For the gene discovery option, which is designed to identify natural enzyme variants, candidate variant sequences are submitted to the platform as a FASTA file.. These variant sequences are pre-selected based on their similarity to the template enzyme, identified through BLAST searches against sequence databases. The mutation space scales exponentially with the number of residues to mutate, and, for this reason, the GDEE platform was created to allow automated and high-throughput generation and testing of thousands of protein sequences.

### Model Generation and Optimization

Homology models are built and optimized by Modeller^20^ with the user-provided enzyme structure serving as a template. Models are built using the methods present in the program. Special restraints may be applied in the modeling phase, such as an optimization radius and an optimization level. The optimization radius defines the subset of amino acid residues selected for geometry optimization. This subset can include only the mutated residues (with an optimization radius of 0) or both mutated and neighboring residues. The optimization radius, measured in angstroms, sets a cutoff distance from the mutated residues to select neighboring atoms for optimization. This optimization applies only to side chains, while keeping the backbone fixed to preserve the protein’s overall folding. The user may also choose optimization and refinement levels to the optimization process. Three optimization levels (0, 1, and 2) are available. Level 0 performs a fast optimization using the *very_fast* schedule and a short MD run, with up to 100 evaluations of the objective function. Level 1 conducts a standard optimization with the *normal* schedule and a slower MD routine, allowing up to 200 iterations. Level 2 carries out an extensive optimization, applying the *slow* schedule and *very_slow* MD mode, with a maximum of 300 objective function evaluations. The platform will create three times the number of models defined by the user for each of the sequences generated in the previous step, allowing to explore structural diversity, and enabling quality assessment and selection of the best models. To prevent inferior quality models from being passed on to the docking step and then delivered to the user, two structural quality assessment methods, namely VoroMQA^23^ and Modeller’s normalized DOPE^24^ are available in the platform, decreasing the amount of manual curation of the resulting models and improving the performance of the platform by avoiding unnecessary docking calculations. All the models generated are individually scored by each of the quality assessment methods and classified into accepted or rejected. Then, only the best models (based on these scores), according to a user defined number of desired models, are passed on to the docking step. While VoroMQA and the normalized DOPE methods are able to identify significant structural problems, their sensitivity falls short when it comes to detecting subtle structural problems. Consequently, some manual curation remains imperative for refining the resulting models, especially in addressing minor structural anomalies.

### Docking and Binding Affinity Calculations

Docking and binding affinity calculations between the ligand and the protein are performed on all of the generated variant models that passed through the acceptance criteria by the programs AutoDock Vina or Smina. Binding energies are obtained by docking of each provided ligand structure. The end-user may choose between Vina or Vinardo scoring functions. The initial ligand position is obtained from the user specified PDBQT file without modifications. The exhaustiveness parameter can be defined by the user and random seeds are randomly generated on each run. The box size and center must be provided by the user. The reference binding affinity (binding affinity of the reference protein (WT)) is also obtained by the aforementioned methods. The protein is considered rigid throughout the binding affinity calculations. Protein structures are automatically processed by AutoDockTools^25^ scripts.

### Data Parallelism

We note that the steps described above can be performed on distributed systems using MPI. As a parallelizable solution, the GDEE platform enables automated high-throughput calculations, significantly enhancing computational efficiency.

### Storage of Data

All data obtained on each step is stored and organized as entries in specific tables in a SQLite database. Data is thus easily accessible to debug the pipeline or to perform extra analysis needed by the end-user, such as filter poses to obtain only those in catalytically relevant orientations.

The database is composed of 7 tables (Evaluations, Measurements, Metrics, Models, Poses, Proteins and Variants). The diagram for the relational model of the database is shown in Figure 2. The table “Proteins” contains the records for each reference protein used. Here we can have information on the name of the protein and the Uniprot ID. The table “Variants” register information on the name of the variant (in enzyme engineering cases, this is the mutation, e.g., A:V220L, and in the gene discovery cases, this contains the sequence identification code present in the FASTA file), its sequence, the directory name where the resulting files are stored and a Boolean to indicate whether it is the wild type (or the reference protein in gene discovery cases). It also has the possibility to add a PDB file and code for each variant if the user desires to. The “Evaluations” table links the variants, models, and poses to an evaluation. In addition to these links, there is also information on the ligand name and file used in each evaluation process, the method utilized for the evaluation step and the resulting PDB file. The “Models” table retains information regarding the variant which the model corresponds to, the software used to create the model, the scores for each model, the resulting PDB file and a Boolean to indicate if the model was rejected or not. The information about the docking results is stored in the “Poses” table, which contains the pose index and the associate energy for each evaluation. The two remaining tables “Metrics” and “Measurements” hold information on the distances that are going to be used for the filtering step. The first one, associate the metric’s name to the metric itself and the other links each pose to the metric and its value.

**Figure 2:**
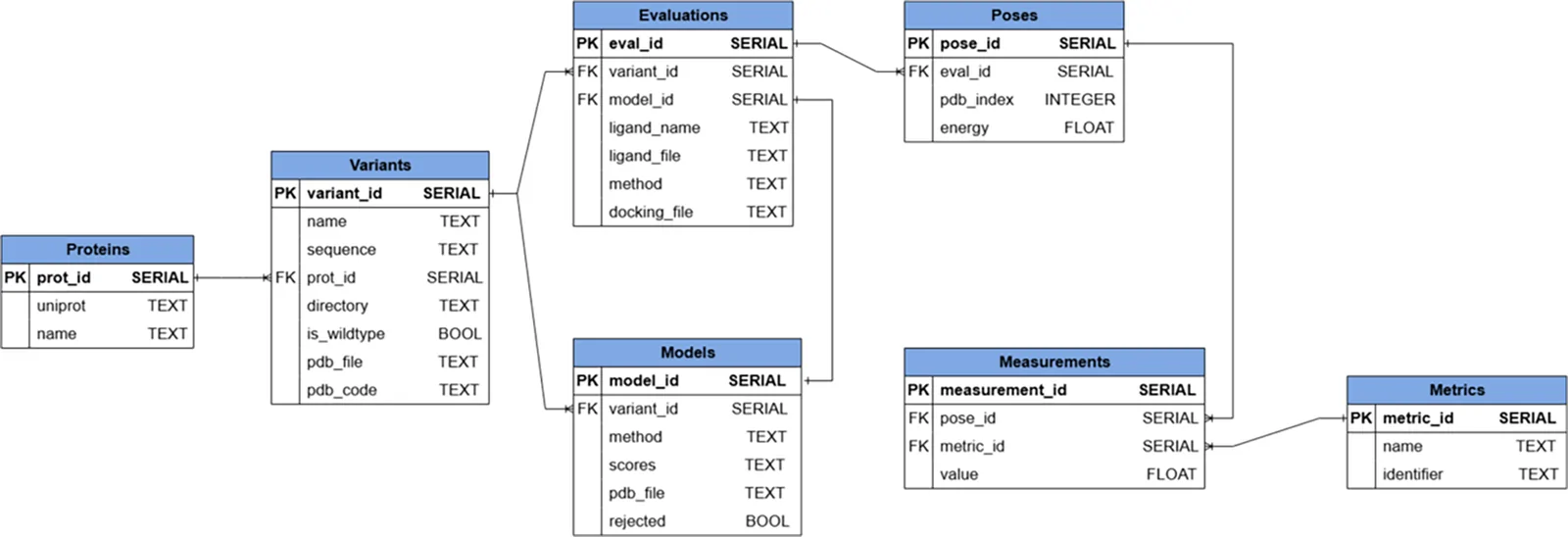
Relational model (entity-relationship diagram) of the platforḿs database. This diagram depicts the logical structure and organization of the database used here, including all entities, their principal attributes, and the relationships among them. Entities are represented as tables containing attributes, with primary keys indicated and foreign key relationships explicitly shown. Relationships between entities—such as one-to-many or many-to-many associations—are indicated using standard ERD notation for cardinality. This model captures how resulting data is integrated, supporting robust data storage, retrieval, and linkage across all steps. See text above, for the explanation.

### Filtering Process and Ranking Step

To ensure that ligands are in a catalytically relevant orientation, the docking results are filtered using distance constraints between atoms from the ligand and the candidate enzyme. The distances are stored in the database mentioned above, so ligand poses relevant to the reaction are easily queried. For cases where more than one pose is accepted in the filtering criteria, the selected ligand pose for each variant sequence is the one with the best binding affinity value.

## Preparation Protocols and Parameters Selection

The full details of preparation protocol recommendations and the parameter selection benchmark are provided in the Supporting Information Sections S1 and S2, respectively.

In this benchmark, we show that using five models for each variant in the docking calculations with an exhaustiveness of at least 100, as the parameters for docking steps in the platform, provide an effective compromise between accuracy and computational cost. These settings have been adopted as the standard configuration in our protocols.

## Re-scoring of Docking Poses with Machine Learning Scoring Functions

The platform has been applied to multiple case studies, with several successes (two published,^18,19^ with others forthcoming) as some less successful outcomes that revealed areas for improvement, particularly in scoring accuracy critical for robust ranking.

The docking step is crucial to evaluate the variants, but while pose generation is reliable, current scoring methodologies limit docking accuracy. To address this, a re-scoring module using machine learning-based (ML) functions has been integrated in the platform. These non-parametric ML methods implicitly capture complex binding interactions without assuming a fixed functional form, allowing direct inference from experimental data.^26–28^ This method has demonstrated improved scoring performance in previous studies.^26–30^ For this, seven ML-based scoring functions (Rf-Score v1,^28^ Rf-Score v2,^30^ Rf-Score v3,^29^ NNScore,^26,31^ PLECrf, PLECnn, and PLEClinear^32^) were selected from the literature for further testing and comparison with classical ones (Vina, Smina, Vinardo and AutoDock4.2 (AD4)^25^). These functions predict p*K_i_*/*d*, so to compare with the others that calculate Δ*G*, we converted the respective results using the following equation, as described in ref 33

$$\Delta G=\mathrm{RT}\ln10^{-pK_{i}/d}$$

### Scoring and Docking Precision

All scoring functions were first assessed for scoring and docking precision using 4482 protein-ligand complexes from the PDBBind v2020 refined set.^34^ Scoring precision measures how accurately a function predicts the affinity of binding poses, while docking precision assesses each scoring function’s ability to identify the correct binding mode. Figures S5 and S6, of Supporting Information, show the results for the scoring and docking precision evaluation, respectively. Regarding scoring, Vina, Smina, Vinardo and AD4 performed similarly. Among ML models, Rf-Score v2 achieved the best performance, whereas PLEC-based functions performed poorly. For the docking precision evaluation, classical functions outperformed ML models. Vina correctly identified 54% of poses within 2 Å of the crystal structure, followed by Smina, Vinardo and AD4 (∼45% each). ML models performed worse (2–17%). These findings suggest that ML-based functions excel at affinity predictions but remain less effective in distinguishing correct poses from incorrect ones, probably due to training primarily on experimental structures. This suggests that the most effective protocol uses AutoDock Vina for docking to identify the best pose, followed by ML re-scoring to enhance binding affinity prediction accuracy. Further details can be found in Section S3 of Supporting Information.

### Evaluating Scoring Within the Scope of Enzyme Engineering

In enzyme engineering, a precise understanding of how mutations influence ligand binding is essential for designing enzymes with enhanced catalytic properties. With this objective in mind, we use 260 pairs (from the pipeline benchmark, see Supporting Information S2) of related proteins to evaluate how well the functions predicted the effects of mutations on binding. For each protein pair (reference and variant), we determined the change in binding free energy (ΔΔ*G*) and compared these predictions to experimental data. A method was considered successful if it correctly predicted the sign of the free energy difference, i.e., whether the mutation improved or reduced the protein’s binding affinity to the ligand.

Figure 3 depicts the percentage of successes, of each scoring function, on predicting the mutation’s effect on binding. In most cases, the ΔΔ*G* values are very small, because the Δ*G* values of reference and variant proteins are very similar and often fall within Vina’s error range. Scoring functions of Vina, Smina, Vinardo, and AD4 correctly predicted approximately 50% of cases, both from X-ray structures and after docking calculations. Among all methods, Rf-Score v2 showed the best performance in the scoring-only, with a success rate around 72% on X-ray structures, and was the only ML-based scoring function whose improvement was clearly above the error range of the other models in this setting. After docking, however, its success rate decreased to 55%, placing it within the error range of the remaining ML-based scoring functions. NNScore also demonstrated an improved success rate, around 60% in both scenarios, however its predictions remained within the error range of the other scoring functions as well. The remaining models performed similarly or worse than the classical scoring functions.

**Figure 3:**
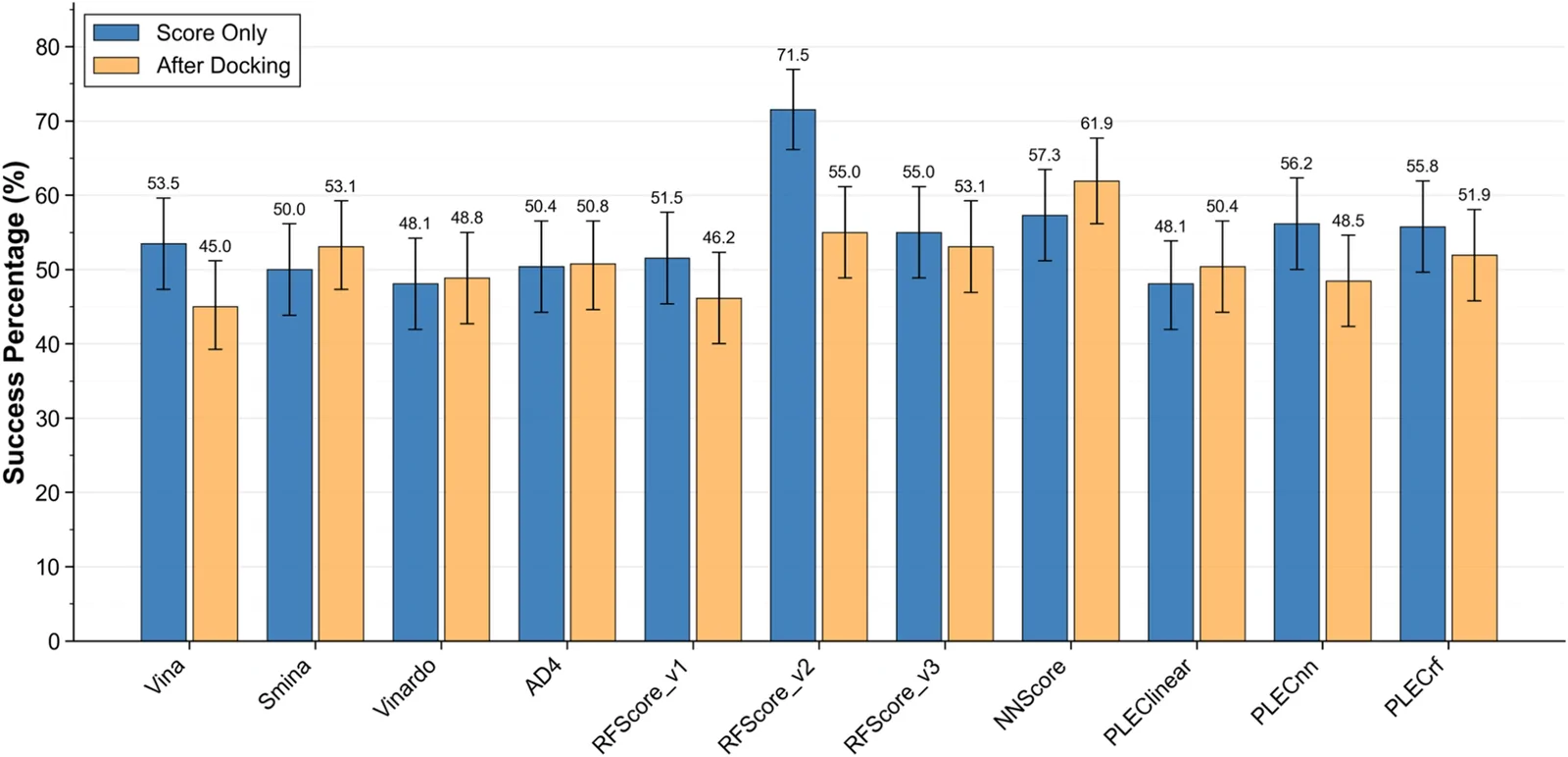
Percentage of successes for each scoring function in predicting the mutation’s effect on binding affinity within 260 of reference-variant pairs. Success is defined as the fraction of cases in which the scoring function correctly predicted the sign of the change in binding free energy (ΔΔ*G*) compared to experimental data. Bars show the observed success rates, with asymmetric error bars representing 95% percentile-bootstrap confidence intervals calculated from 10000 resamples of the reference-variant pairs with replacement. Results are shown for scores calculated using both X-ray crystal structures (blue) and structures after ligand re-docking into the protein’s active site (yellow), based on the lowest-energy pose identified by Vina. Classical scoring functions including Vina, Smina, Vinardo, and AD4 achieved approximately 50% success rates across both structural contexts. Among machine learning models, Rf-Score v2 performed best, with success rates of 72% on X-ray structures, and was the only model whose performance was clearly above the error range of the other ML-based models. After docking, RF-Score v2 achieved 55%, which fell within the error range of other models. NNScore also showed improved rates near 60%, however, its predictions remained within the error range of other models as well. Other ML models displayed similar or reduced accuracy compared to classical functions. This assessment underscores the variations in predictive accuracy introduced by mutation effects and structural modeling, informing the selection of the best scoring approaches for enzyme engineering applications.

## Stacking Ensemble of Scoring Models

Building on our finding that certain machine learning-based scoring functions improve binding affinity predictions, we explored whether combining these models into an ensemble could further enhance performance. We employed stacking generalization as the ensemble method, integrating the scoring functions studied earlier to create a metamodel in an attempt to enhance scoring performance of the GDEE platform.

We evaluated protein-ligand affinity predictions using the refined PDBBind 2020 dataset in its leak-proof split version.^35^ This split was specifically designed to reduce information leakage across the three sets, train, validation and test, by minimizing structural similarity between complexes and between related structures in any of these sets. All model development, hyperparameter optimization, and model selection were restricted to the training and validation sets only. The test set was kept completely untouched until final evaluation to provide an unbiased estimate of generalization.

### Exploring all Model Combinations

To evaluate whether model diversity improved performance, we tested multiple base-model combinations drawn from the available scoring functions. The output predictions of each candidate subset of base models were used as the input for the second-level learner in the stacking ensemble framework. We considered combinations ranging from single base models to larger subsets, allowing the data to determine whether a smaller, more selective ensemble outperformed the full stack.

The stacking framework used a second-level meta-model to combine the outputs of the selected base models. We used linear regression as the default meta-learner because it provides a simple, interpretable baseline and often performs well when the base learners already encode complementary information.^36^ In addition, we evaluated alternative meta-models, including Ridge regression, Lasso, Elastic Net, and other candidate regressors, to determine whether regularization or nonlinearity improved performance. These results are not shown here.

To prevent target leakage during stacking, we used out-of-fold predictions within cross-validation, rather than using in-sample predictions. The final meta-model was trained on these out-of-fold predictions, while validation performance was used to compare base-model subsets and meta-model choices. This setup ensured that the validation set served as the model selection set and not as the final benchmark.

Hyperparameters were optimized using grid search on the training data only, with cross-validation performed inside the training set. Candidate hyperparameter grids were defined for each meta-model, including regularization strength for linear models and kernel or tree parameters for nonlinear learners. The validation set was then used only to compare the best configurations emerging from the training optimization. This avoided using the test set during any form of tuning and preserved an unbiased final evaluation.

Metamodel optimization and model combination benchmarking are detailed in Supporting Information Section S3.1.

The best combination, consisting of a linear regression model of five base models, is detailed in Table 1, along with their respective weights. Notably, Rf-Score v2 carries significantly more weight than the others, reflecting its superior individual performance. This metamodel was then applied to re-score protein-ligand complexes, and its performance was compared against other models.

**Table 1:** Composition and Relative Weights of the Five Base Models in the Optimized Stacking Ensemble Metamodel^a^

Base model	Weight
β0	2.259
Rf-Score v1	–0.903
Rf-Score v2	2.206
PLECrf	–0.178
PLEClinear	–0.057
Vinardo	0.056

a This table summarizes the individual base models selected for the ensemble and their assigned weights reflecting their contribution to the final predictive model. Notably, Rf-Score v2 carries significantly greater weight than other models, consistent with its superior standalone performance. The weighted combination effectively integrates diverse predictive strengths and was subsequently applied to re-score protein-ligand complexes, demonstrating enhanced accuracy compared to individual scoring functions.

Figure 4 presents histograms comparing predicted and experimental binding affinities, both for the X-ray structures and after re-docking ligands using AutoDock Vina. The metamodel outperformed all individual scoring functions. However, its performance remained within the error range of RF-Score v2, which showed a comparable MSE (see Figure S5 in Supporting Information). The metamodel nevertheless achieved a higher *R* ^2^, which may indicate its superior ability to explain the variance in the data.

**Figure 4:**
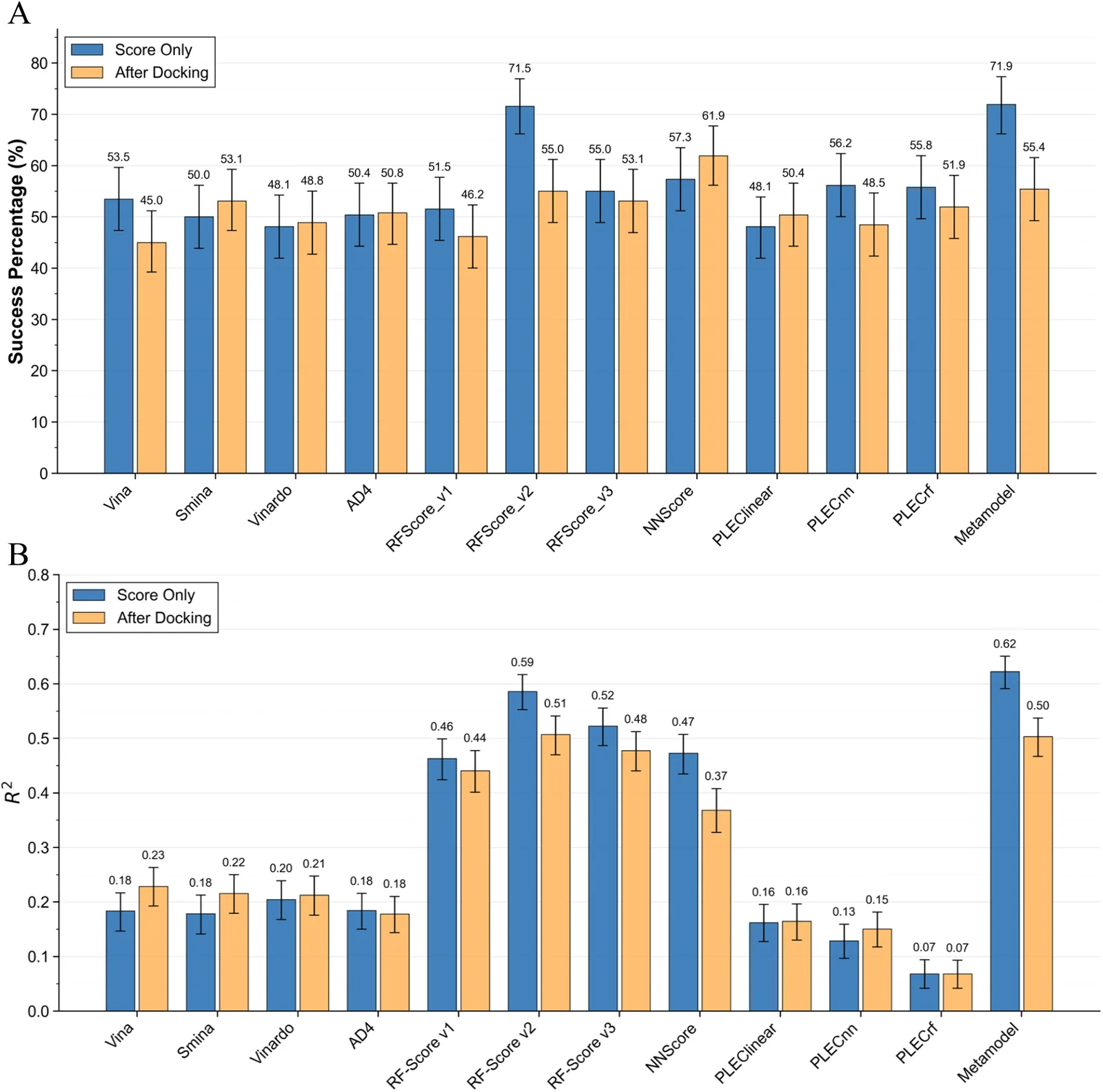
Histogram comparison of predicted versus experimental binding affinity metrics for the stacking validation set. (A) R^2^ between predicted and experimental values for X-ray crystal structures (blue) and ligand re-docked structures using AutoDock Vina (yellow), based on the lowest-energy pose identified by Vina. (B) Percentage of successes in predicting the sign of the mutation’s effect on binding affinity (ΔΔ*G*) for the same structural contexts. Bars show the observed success rates, with error bars representing 95% percentile-bootstrap asymmetric confidence intervals calculated from 10000 resamples of the reference-variant pairs with replacement. The metamodel outperformed all individual scoring functions, exhibiting superior *R* ^2^, although its performance remained within the error range of RF-Score v2, showed comparable MSE (see Figure S5 in the Supporting Information). For mutation subsets within the database, the metamodel achieved similar performance with the best individual model (Rf-Score v2), reaching 71% on X-ray structures and 55% post-docking.

When analyzing the mutations within the database (Figure 4B), the metamodel showed a similar performance compared with Rf-Score v2’s success rate (the best individual function for both cases). While the metamodel is clearly superior to most models in predicting binding affinities, its advantage over Rf-Score v2 is less evident when comparing the effects of mutations.

While comparing the Gibbs free energies between reference and variant enzymes from X-ray structures offer valuable insights, this work focuses specifically on evaluating scoring functions within the platform’s full pipeline. Unlike previous analyses limited to docking, the platform also predicts mutant enzyme structures, enabling an integrated assessment that reflects scoring performance on predicted rather than experimental models.

For this evaluation, the platform was applied to 187 reference proteins (corresponding to the 260 pairs of reference-variant proteins, as some references have multiple variant counterparts) to generate the respective variants. All scoring functions, except Vina (already implemented), were then used to re-score the resulting complexes. This approach provides a holistic evaluation of scoring functions across the platform workflow, though based on fewer complexes than the analysis in Figure S5, since not all proteins had corresponding variants. Consequently, direct comparison between the two evaluations is not feasible. Results regarding docking precision and comparing experimental and predicted binding affinities are presented in the Supporting Information (Figures S18 and S16, respectively). All three versions of Rf-Score continued to provide high predictive performance regarding *R* ^2^.

Although predicting quantitative changes in binding affinity between mutants is desirable, enzyme engineering often requires a more pragmatic output: prioritizing variants that are more likely to improve ligand binding for experimental testing. Under our assumption that improved binding can contribute to improved catalysis, Figure 5 evaluates this qualitative prioritization by measuring the percentage of mutations for which each scoring function correctly predicts whether binding is improved or reduced. RFScore v1 showed the highest success rate, with correct predictions in nearly 54% of cases for the 260 reference-variant pairs. This performance is only modestly above random baseline and should therefore not be interpreted as supporting binary success/failure prediction for individual mutations. Instead, it indicates the current practical limit of this high-throughput structure-based workflow, where uncertainty can accumulate from protein structure prediction, rigid-receptor docking, pose selection, and scoring. Nevertheless, even modest enrichment can be useful for reducing large candidate libraries before experimental testing, especially when combined with biochemical knowledge and active-site inspection.

**Figure 5:**
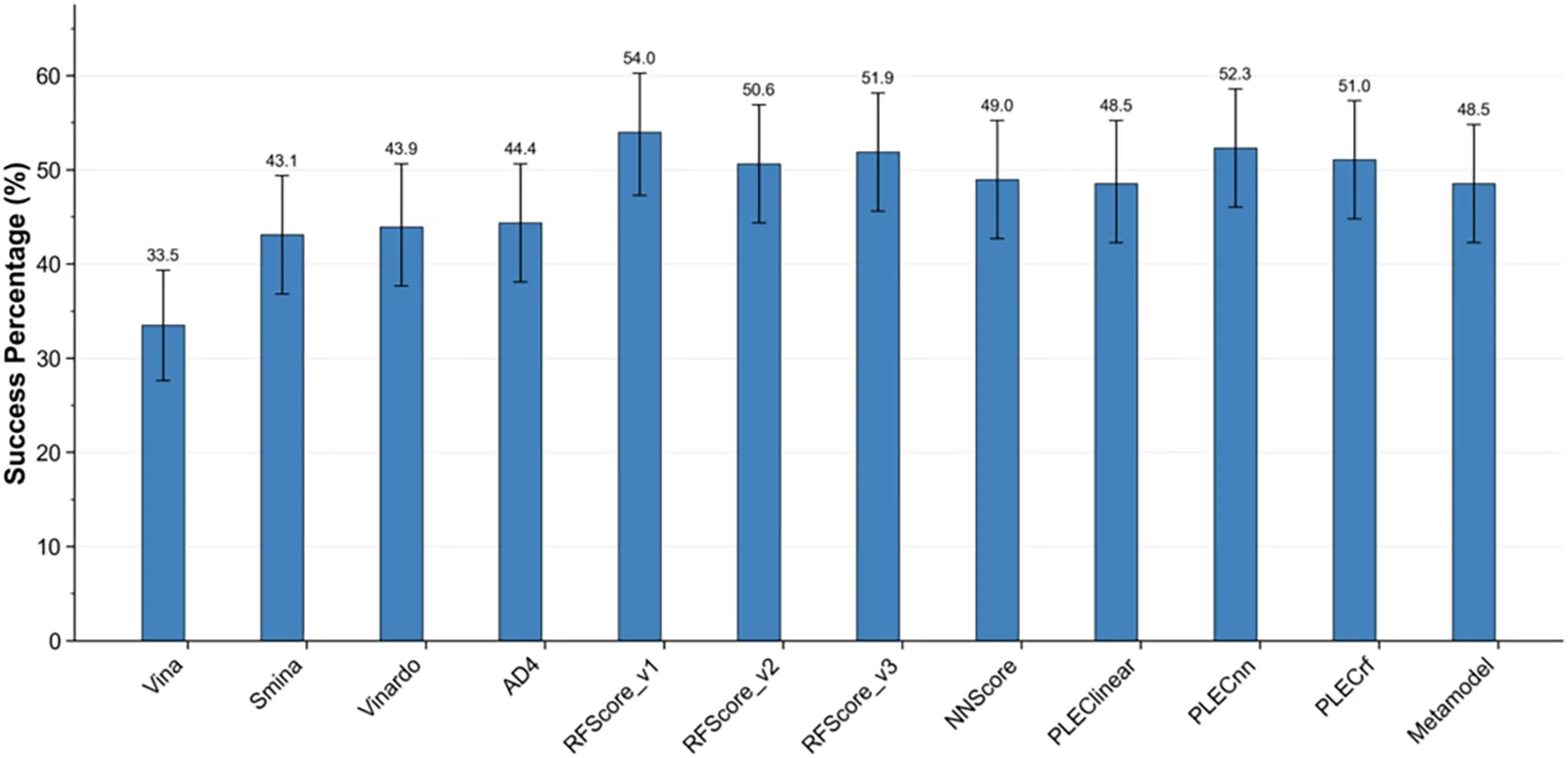
Percentage of successes for predicting mutation effects on binding affinity across scoring functions using the platform’s pipeline in the stacking validation set. Success is defined as correctly predicting the qualitative change in binding affinity (i.e., the sign of ΔΔ*G*) based on experimental measurements. Predictions consider the lowest-energy pose obtained by Vina. Bars show the observed success rates, with error bars representing 95% percentile-bootstrap asymmetric confidence intervals calculated from 10000 resamples of the reference-variant pairs with replacement. The histogram illustrates the comparative performance of multiple scoring functions on the 260 reference-variant pairs, highlighting variations in predictive accuracy. RFScore v1 achieved the highest success rate, while other scoring functions exhibited diverse results, potentially influenced by structural modeling uncertainties during protein structure prediction.

The reduced performance of the stacking ensemble within the full pipeline likely reflects the accumulation of uncertainty across multiple upstream steps. In the first benchmark setting, scoring is evaluated on X-ray or re-docked structures, which largely isolates the affinity-prediction task. In contrast, the full GDEE workflow relies on homology-modeled receptor structures generated without the ligand present, followed by rigid-receptor docking and pose selection, all of which can introduce small structural deviations that propagate into the final score. In addition, the machine-learning scoring functions were trained mainly on experimental protein-ligand complexes, so their input distribution may differ from the enzyme-engineering scenarios considered here. Together, these factors likely explain why the ensemble retains good performance in the first benchmark tests but shows reduced robustness when embedded in the complete pipeline.

The metamodel was selected as the preferred scoring function for rescoring docking poses because it showed the most consistent performance across the different evaluation settings. In the initial benchmarking, performed independently of the complete platform pipeline, it achieved the highest *R* ^2^ while maintaining a competitive MSE relative to the individual machine learning models, indicating that it captured the experimental trends more effectively than any single scoring function.

Although these improvements were modest, they are particularly relevant for ΔΔ*G* prediction, where most mutations produce only small changes in binding free energy and the predicted Δ*G* values for the reference and variant proteins often fall within the intrinsic uncertainty of binding estimators. Under these conditions, even small gains in predictive accuracy and ranking can improve mutation prioritization. This behavior is consistent with the well-established advantage of ensemble approaches, which combine complementary predictive signals and often outperform individual models.^37,38^ Nevertheless, the platform remains fully configurable, allowing users to choose the metamodel, any individual machine learning scoring function for rescoring docking poses, or the original Vina or Vinardo scoring function from the docking step.

## Conclusions

Enzyme engineering has become a cornerstone in numerous fields, ranging from pharmaceuticals and biocatalysis to sustainable energy and environmental remediation. The growing demand for custom-designed enzymes with enhanced stability, specificity, and catalytic efficiency underscores the need for advanced tools that can accelerate and optimize the engineering process. In this work, we introduce a comprehensive computational platform specifically designed to address this need, offering a powerful suite of tools for identifying natural enzymes capable of catalyzing specific reactions and to facilitate the *in-silico* optimization of enzyme properties.

The case studies referenced here exemplify the GDEE platform’s potential for broad applications in synthetic biology, rapidly identifying and optimizing enzyme variants for specific functions, demonstrating its potential as a powerful tool in metabolic engineering.

Additionally, this study highlights the potential of ML scoring functions to significantly enhance the accuracy of binding affinity predictions in protein-ligand complexes. While these models show great promise in predicting binding affinities, their effectiveness in selecting the optimal pose from docking calculations is limited. This limitation likely stems from their training on experimentally determined structural data, which enables them to learn correct poses but restricts their ability to recognize incorrect ones.

Given these insights, the most effective protocol involves performing docking calculations using AutoDock Vina, which demonstrated superior accuracy in identifying the best pose. Then, re-scoring this pose with a ML scoring function improves the predictive accuracy of binding affinity. This combined approach leverages Vina’s strength in pose selection and the precision of machine learning models for affinity prediction.

To incorporate these findings, re-scoring with the ml-based scoring function studied here has been implemented, as an optional step in the GDEE platform after the filtering phase, allowing users to re-score the poses identified by Vina and thereby improve ranking precision.

## Supplementary Material



## Data Availability

The source code for the GDEE platform is available at https://github.com/protein-modelling-itqb/gdee, and all associated data can be accessed at 10.5281/zenodo.17553400. Both are provided under the Creative Commons Attribution-NonCommercial-ShareAlike 4.0 International License.

## Notes

Parameter selection benchmark (Figures S1–S3); metamodel combination benchmark (Figure S4); and all plots from the re-scoring study (Figures S5–S19) (PDF)
