# Supplementary material for: GDEE: A Structure-Based Platform for Gene Discovery and Enzyme Engineering
Source: J Chem Inf Model. 2026 Sep 22;66(19):12205–15. doi: 10.1021/acs.jcim.5c02688 (PMC13663853; doi:10.1021/acs.jcim.5c02688)
Supplement: Supplementary file 1 [file ci5c02688_si_001.pdf]

Supporting Information

GDEE: A Structure-Based Platform for Gene Discovery and Enzyme Engineering

Caio S. Souza[$], João P.G. Correia[$], Isabel Rocha, Diana Lousa, Cláudio M. Soares[*]

Instituto de Tecnologia Química e Biológica António Xavier, Universidade Nova de Lisboa, Av. da República, 2780-157 Oeiras, Portugal

[$]Both authors contributed equally to this work

[*]Corresponding author

S1. PREPARATION PROTOCOLS

S1.1. Gene Discovery

The standard preparation protocol for gene discovery begins with selecting a template structure that provides an appropriate protein fold for the *in-silico* search for enzymes capable of catalyzing the target reaction. Notably, the template does not need to catalyze the target reaction itself; it simply needs to offer a compatible structural framework. Once a suitable template is identified, the ability of the active site to accommodate the reaction substrate or intermediate is typically assessed by generating a model of the enzyme–ligand complex. This can be accomplished either through ligand-aware structure prediction methods, such as AlphaFold3 [15], which directly predicts the structure of protein–ligand complexes, or by performing conventional docking calculations. Both approaches yield valuable insights into substrate binding and guide the establishment of the metrics that will be used in the filtering criteria to be employed in the platform steps. In particular, ligand-aware modelling methods can be useful for selecting or refining an appropriate template for use within GDEE, allowing variant structures to be modelled while preserving, as much as possible, the relevant active-site geometry associated with ligand binding. This approach is especially valuable in cases where ligand binding induces conformational changes in the protein, as using a ligand-bound template can help maintain a more realistic active-site arrangement. However, it should be noted that the Modeller optimization step does not include the ligand and therefore does not account for ligand–protein interactions during side-chain energy minimization.

Subsequently, candidate enzyme sequences are identified by running BLAST searches of the template sequence against a comprehensive database such as SwissProt (Boutet et al., 2007). For reliable homology modeling, BLAST hits are filtered to retain sequences with at least 30% identity and 60% coverage relative to the template. The resulting list of candidate variants,

along with the template structure and the substrate or intermediate structure, are then submitted to the platform's Gene Discovery feature for ranking and further evaluation.

S1.2. Enzyme Engineering

For enzyme engineering applications, it is important to gather information in the literature about the reaction mechanism of each enzyme to help choose the best combination of mutation sites in the protein and the ligand to be used in the process. Whenever possible, experimentally determined structures should be used; otherwise, comparative or ligand-aware models can be generated. With the advent of AlphaFold3 [15], predictions of protein–ligand complex structures are now accessible, providing valuable template models for enzymes and their bound substrate. These approaches yield valuable insights into substrate binding and guide the establishment of the metrics that will be used in the filtering criteria to be employed in the platform steps, as discussed above. The structure of the enzyme to be engineered and the structure of the reaction substrate/intermediate are submitted to the Enzyme Engineering feature of the GDEE platform, as well as the list of residues to be mutated, where enzyme mutants are generated and then evaluated.

S1.3. Platform Parameters and User Flexibility

In both protocols, after running the platform pipeline, filters are applied to the results to ensure that ligands are in a catalytically relevant orientation. In the end, a manual inspection is suggested to carefully analyze the results. For the platform parameters, we usually use an optimization radius of 8 Å (matching Vina's interaction cutoff). As the acceptance thresholds for the modeling step, we follow the threshold proposed by the authors of each method (>0.4

for VoroMQA, < –1 for normalized DOPE). An exhaustiveness value of 200 is typically used unless computational resources are limited (never below 100) and five models are selected for the docking step (see parameter selection section). However, all these parameters and steps can be fully adjusted by the user to accommodate particular needs or specific project requirements.

S2. PARAMETERS SELECTION

A parameterization procedure was conducted to identify suitable values for the main parameters used at each step of the pipeline. The benchmark was derived from the PDBBind v2018 refined set, which contains experimentally characterized protein–ligand complexes with available X-ray structures and measured binding affinity constants. Although PDBBind is not specifically an enzyme-engineering dataset, it provides a structurally and experimentally grounded resource for evaluating the docking and scoring components of the workflow. In particular, it enables systematic assessment of whether the pipeline can capture changes in ligand binding associated with sequence variation (mutation).

To construct the reference/variant pairs, all complexes in the dataset were first screened for similar proteins with minor sequence differences and comparable ligands, and the resulting candidate pairs were then processed to remove duplicates. We considered a variant to be a sequence containing at least one mutation in the active site, defined here as all residues within 6 Å of the ligand, relative to a given reference sequence. This procedure yielded 320 pairs of reference/variant complexes, of which 60 were excluded due to incompatible atom types for

AutoDock4.2 or were considered outliers with an absolute difference greater than 10 kcal mol^{-1} when compared with the experimental values, resulting in a final dataset of 260 pairs. From these, 10,170 variant models (conformations) were generated, and more than 300,000 docking calculations were performed. Protein and ligand input files for docking were prepared using AutoDockTools scripts. For each complex, the docking box was centered on the crystallographic ligand position, and the box dimensions were defined to fully contain the ligand with an additional margin of 2 Å in each direction, ensuring that the ligand-binding region was adequately sampled.

The enzyme-engineering pipeline was then applied to all benchmark entries, using different algorithms and parameter combinations. This allowed us to evaluate the contribution of individual workflow steps, compare scoring functions, and determine parameter values that provide an appropriate balance between binding-affinity sampling, pose prediction, and computational efficiency.

S2.1. Selection of the docking program and scoring function

First, it was crucial to select the appropriate docking program and scoring function for the pipeline. To this end, we used AutoDock Vina, Smina and AutoDock4, each with their respective scoring functions, and Vinardo [25] scoring function (within Smina program), to re-dock each ligand's crystal structure to the corresponding protein.

In enzyme engineering, a precise understanding of how mutations influence ligand binding is essential for designing enzymes with enhanced catalytic properties. With this objective in mind, for each reference/variant pair, the predicted change in binding free energy (ΔΔG) was

calculated and compared with the experimentally derived change in binding affinity. A method was considered successful (a "hit") if it correctly predicted the sign of the free energy difference, i.e., whether the mutation improved or reduced the protein's binding affinity to the ligand. This analysis was used to evaluate the ability of the workflow to predict mutation-associated effects on ligand binding. Importantly, this benchmark does not directly assess catalytic activity. Rather, it tests a specific component of the enzyme engineering problem: whether the platform can identify mutations that are likely to improve ligand binding, This is most relevant for engineering applications in which substrate, intermediate, product, cofactor, or inhibitor binding, as well as productive ligand orientation, are expected to contribute to the desired functional outcome.

Figure S1 shows a histogram comparing the percentage of successful predictions made by each scoring function when classifying mutation effects on binding affinity. Figure S1 shows the percentage of successful predictions referred to as Hits. Among the methods tested, Vina demonstrated the best performance, achieving the highest percentage of successful predictions when considering the pose with the lowest energy or the lowest RMSD for each complex. These results suggest that Vina is a more effective solution for evaluating the enzyme engineering process. However, it is important to note that the $\Delta\Delta G$ values obtained are relatively small, often falling within the typical error margins associated with docking programs. This means that the differences in binding free energy predictions between the reference and variant proteins are subtle and may not be fully captured by the computational methods used. Despite these limitations, the trends observed in our analysis suggest that Vina provides valuable insights, achieving the best performance in the context of enzyme

engineering. Based on these findings, Vina has been selected as the scoring function in our platform.

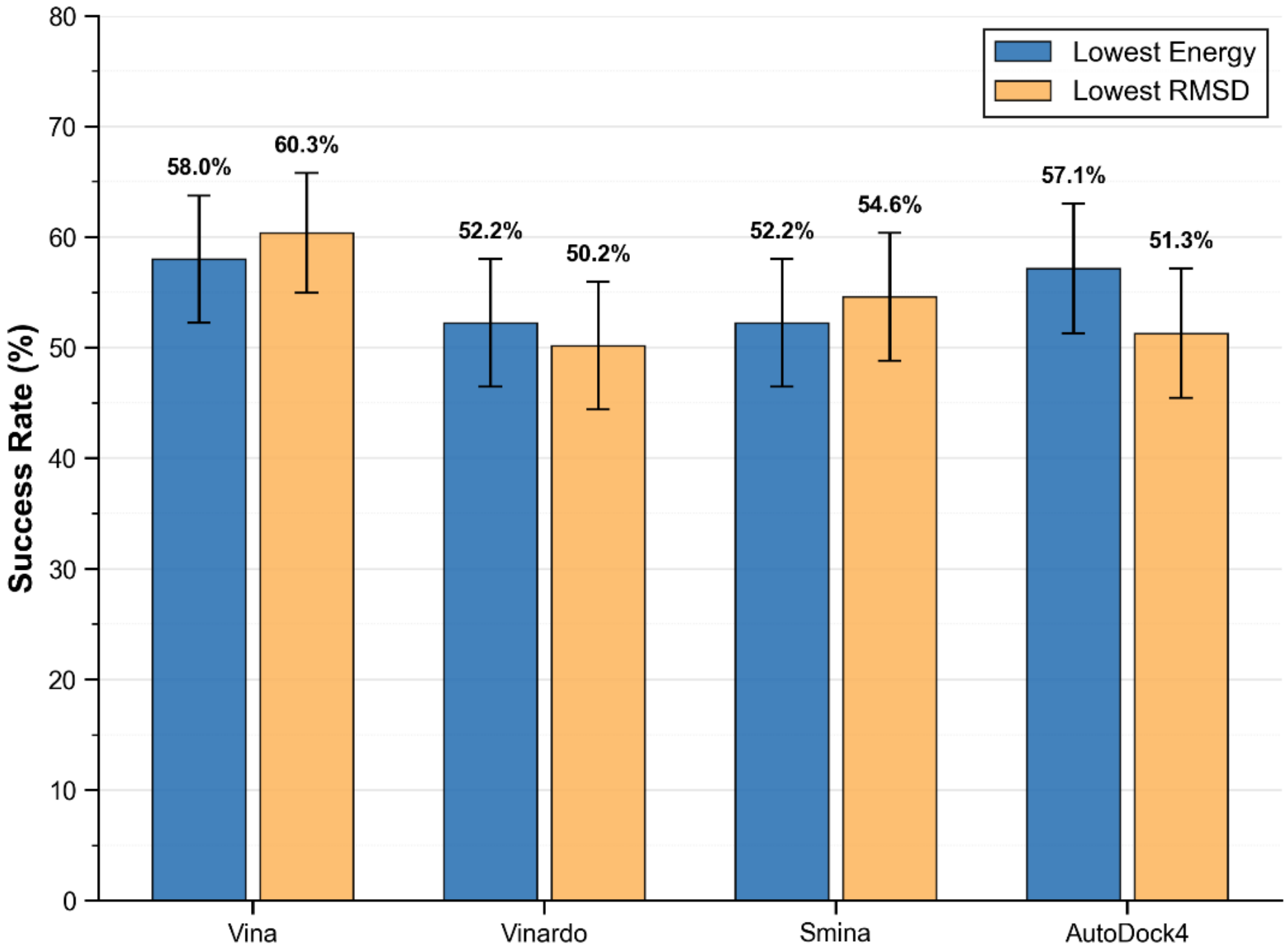

Figure S1 - Percentage of successes for each scoring function in predicting the mutation's effect on binding affinity. Success is defined as the fraction of cases in which the scoring function correctly predicted the sign of the change in binding free energy (ΔΔG) compared to experimental data. For each protein, in blue we considered the docking poses with the lowest energy and in yellow the docking poses with the lowest RMSD. Bars show the observed success rates, with error bars representing 95% percentile-bootstrap asymmetric confidence intervals calculated from 10000 resamples of the reference-variant pairs with replacement. Among the tested approaches, the Vina scoring function demonstrated the highest accuracy in both selection criteria.

A

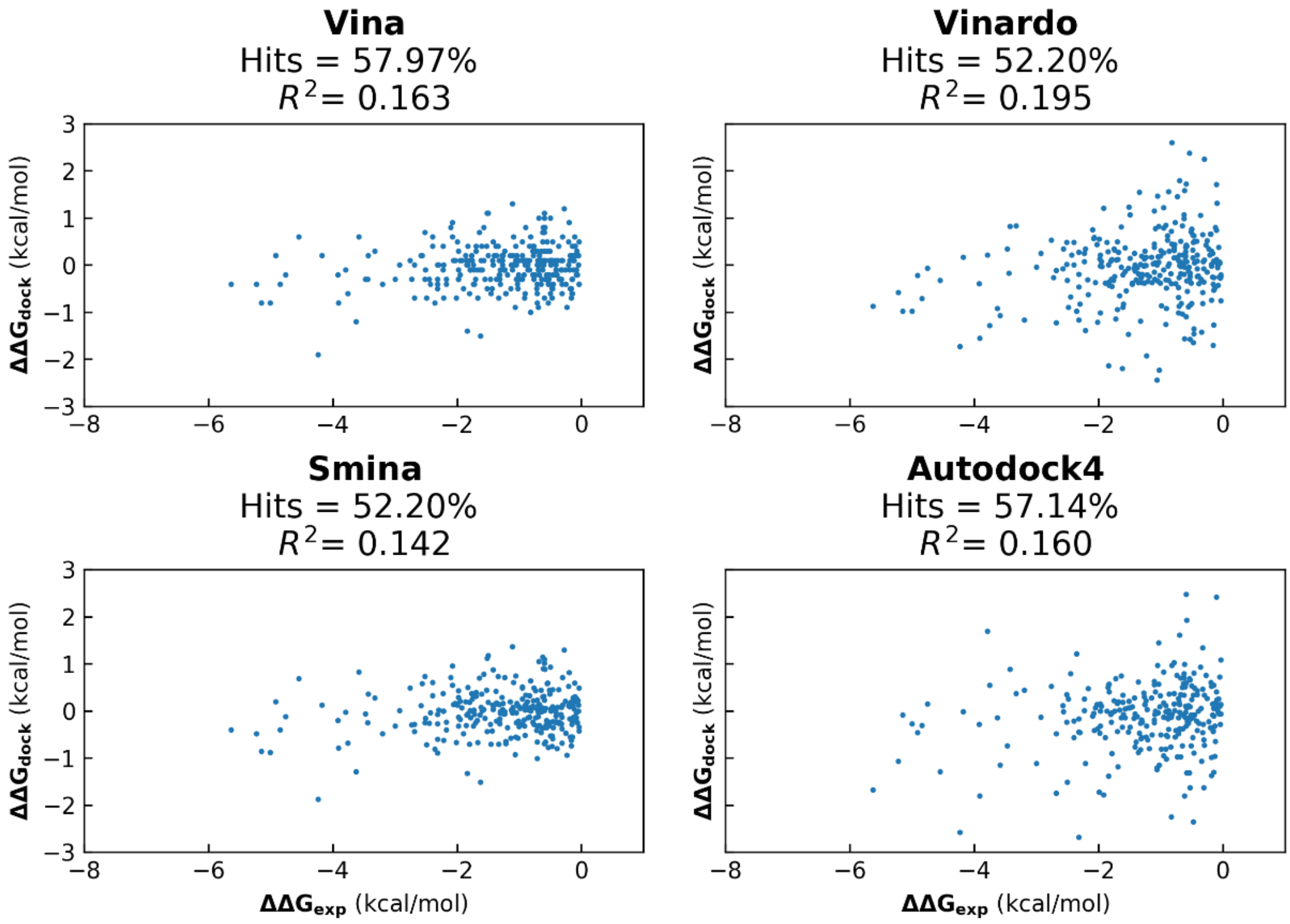

B

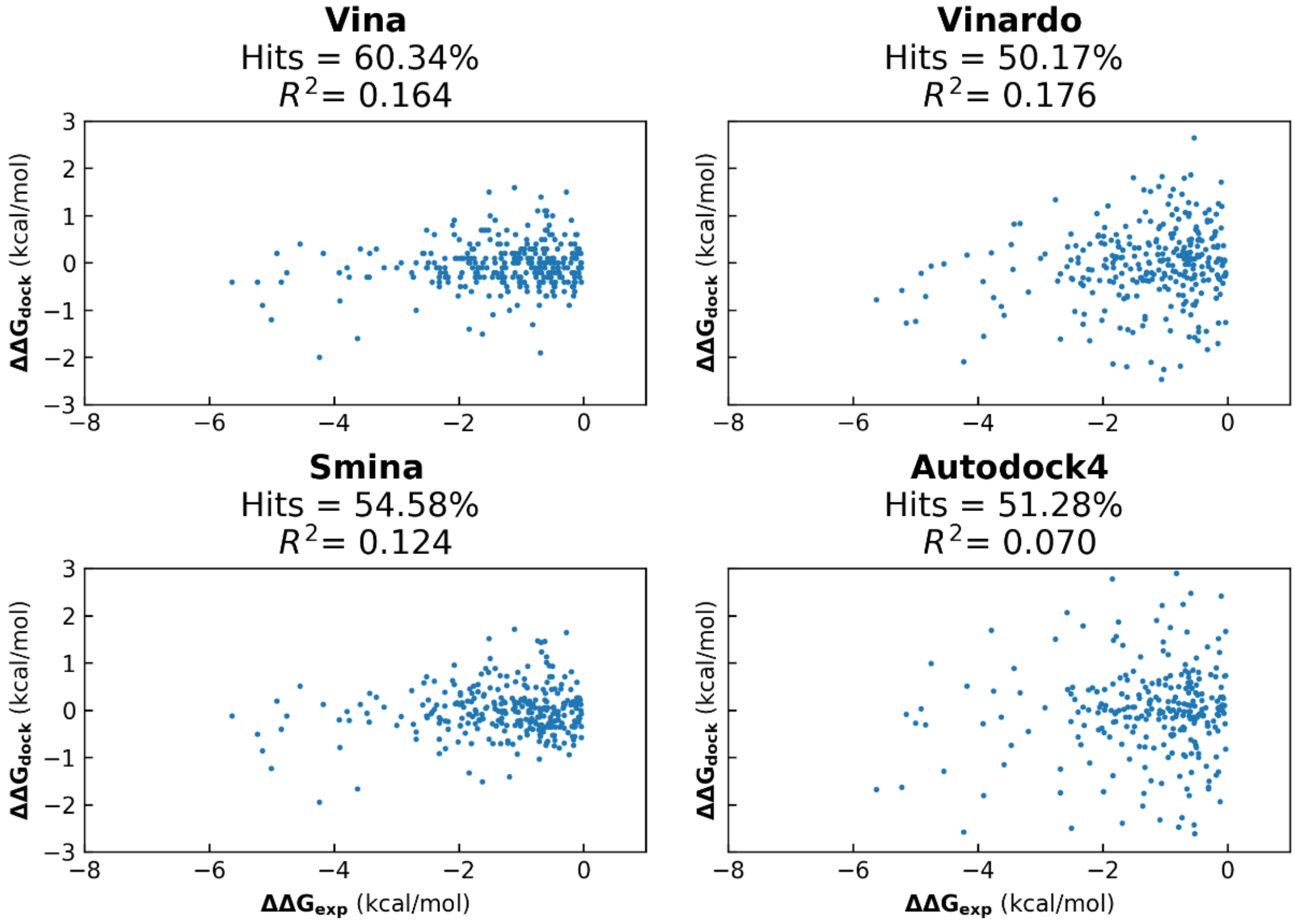

Figure S2 - Scatter plots comparing predicted ΔΔG to experimental ΔΔG. Hits – Percentage of successes on predicting the mutation's effect on binding. Structures from PDBbind v2018 database. For each protein, in (A) we considered the docking poses with the lowest energy and in (B) the docking poses with the lowest RMSD. Among the tested approaches, the Vina scoring function demonstrated the highest accuracy in both selection criteria.

S2.2. Selection of exhaustiveness and number of models

After selecting the docking software and scoring function, it was necessary to determine the appropriate values of exhaustiveness and the number of models used in the docking step. Figure S2 presents heatmaps showing the energy distribution of docking solutions across the tested parameter space (exhaustiveness and number of models), corresponding to different numbers of models used. Each heatmap column represents the energy distribution for a fixed exhaustiveness value. In these heatmaps, a reduction in variance is observed when increasing the number of models from one to five. However, beyond five models, no significant further reduction is observed, indicating that statistical sampling is effectively achieved at five models.

This convergence of variance with multiple models is further supported by the data in Figure S3 (bottom plot), reinforcing the conclusion that five models provide the right balance between comprehensive conformational sampling and computational efficiency.

Figure S3 (top plot) complement these findings, showing that increasing the exhaustiveness parameter from 100 to 300 yields only a minor decrease (~0.2 kcal/mol) in average binding energy. This suggests the docking process has essentially converged within this exhaustiveness range.

Based on these results, we recommend using five models and an exhaustiveness of at least 100 as the parameters for docking steps in the platform. These settings provide an effective compromise between accuracy and computational cost and have been adopted as the standard configuration in our protocols.

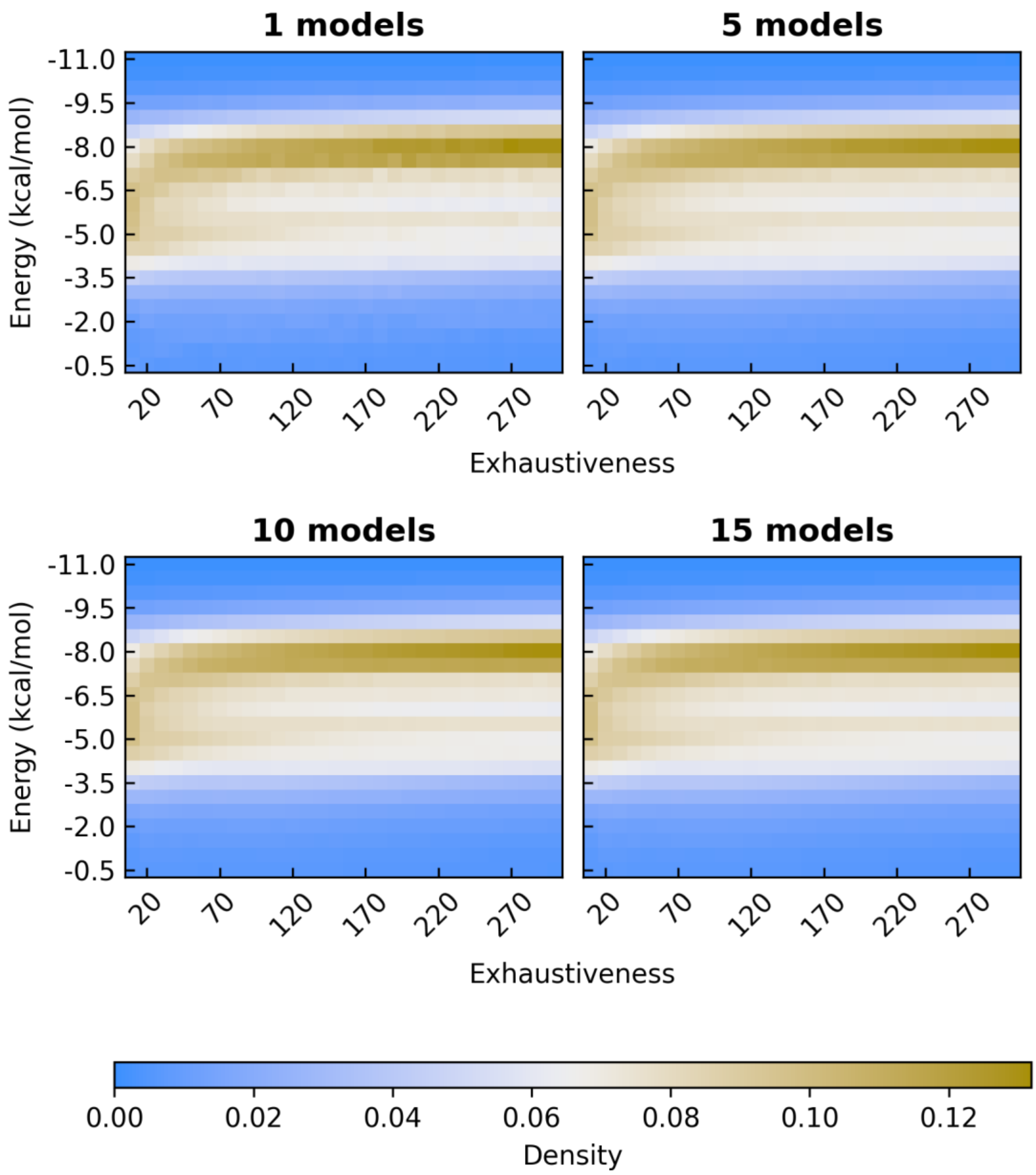

Figure S3 - Heatmaps showing the energy distribution of docking solutions for varying number of models used across different exhaustiveness values. Each heatmap column represents the energy distribution for a fixed exhaustiveness value. Color intensity within each column indicates the density of energy values, with higher density represented by dark golden yellow. Noticeable, differences in energy distribution and reduced noise are observed when the number of models increases from one to five. Beyond five models, further increases do not result in visible improvements.

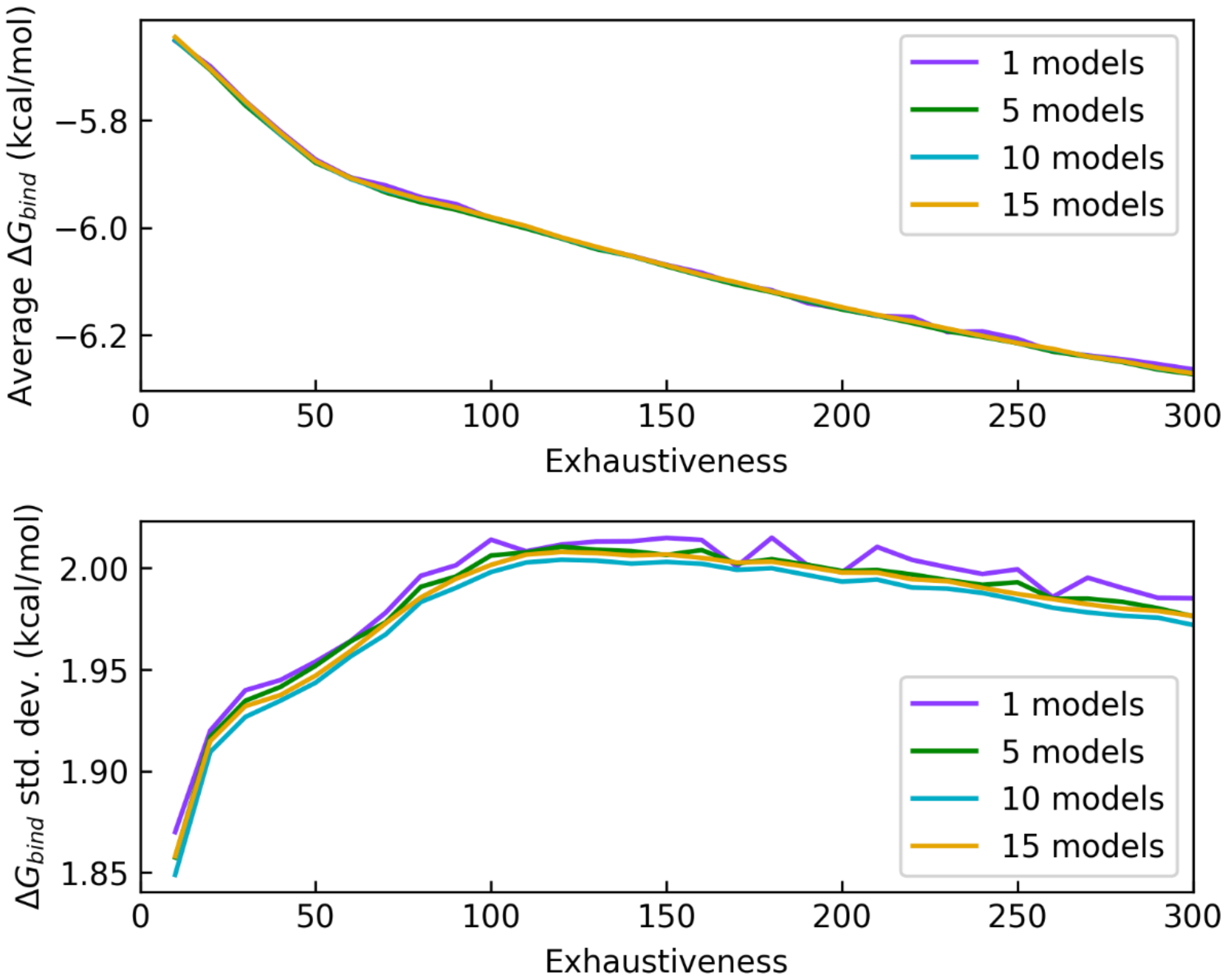

Figure S4 - Comparison of average binding affinity (top plot) and standard deviation of binding affinities (bottom plot) across exhaustiveness values for different number of models used. The bottom plot, clearly demonstrates a smoother standard deviation curve when using more than one model, compared to results from a single model, indicating improved sampling and lower noise.

S3. RE-SCORING OF DOCKING POSES WITH MACHINE LEARNING SCORING FUNCTIONS

S3.1. Exploring all model combinations

We systematically evaluated all candidate combinations of base models using validation MSE as the primary selection criterion. Across the tested subsets, performance varied substantially, indicating that not all scoring functions contributed equally to the stacking ensemble. In general, compact ensembles composed of the strongest and most complementary base models performed better than very large combinations that included redundant or weak predictors.

Figure S4 illustrates the correlation between the number of models and performance metrics (MSE and R^2). Each data point represents the metric values achieved for the best meta-learner and combination of a given number of models. The results demonstrate that increasing the number of models from one to five yields improvements in both metrics. However, beyond five models, further increases result in only small changes in the performance metrics, indicating that additional models offer minimal benefit. For efficiency, using more than five models is not justified, as the gains in predictive performance become limited.

The best-performing subset on the validation set consisted of a linear regression model with RFScore v1, RFScore v2, PLEClinear, PLECrf, vinardo, which achieved a validation MSE of 2.397 and a validation R^2 of 0.570.

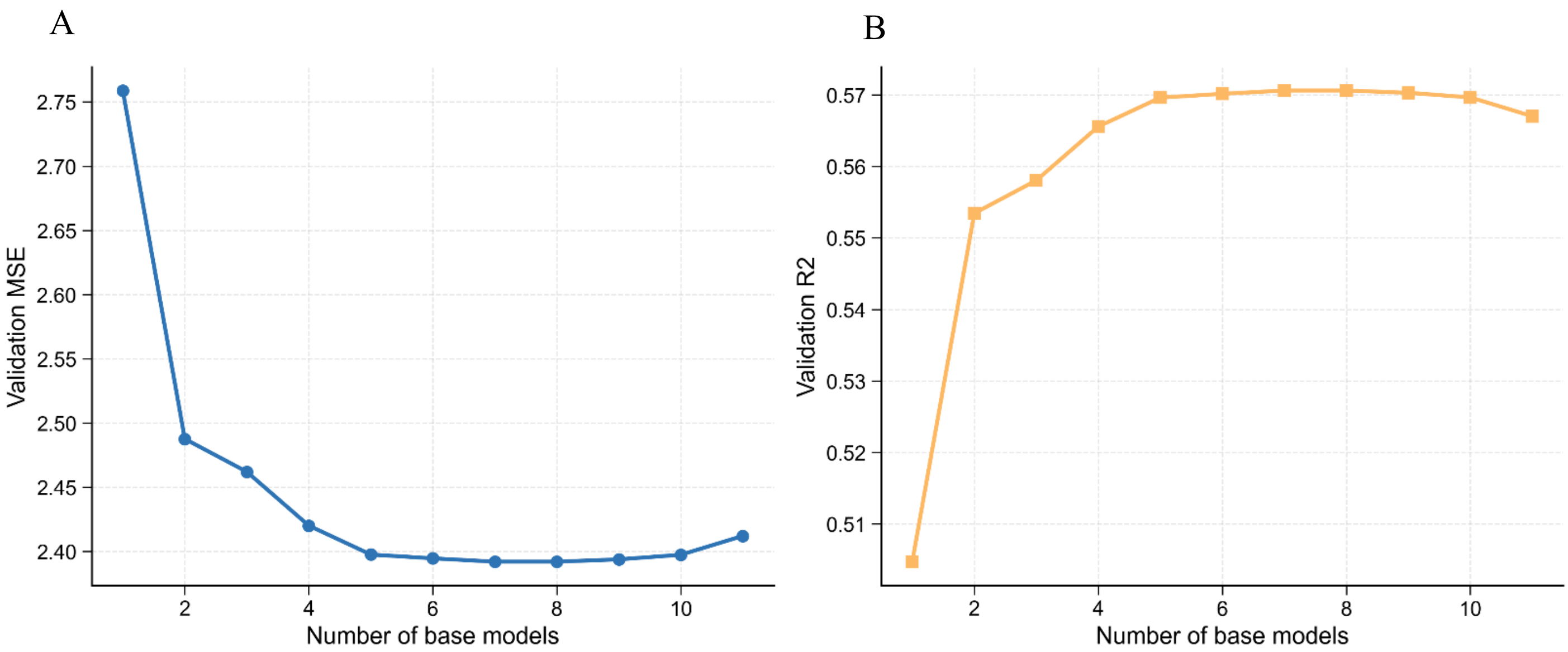

Figure S5 – Relationship between the number of base models used in the stacking ensemble and predictive performance metrics. Plotted are the best achieved MSE (A) and R^2 (B) for each model combination size, evaluated on the validation set. Each data point corresponds to the best combination of base models yielding the best performance for that given number of models. The results reveal a significant improvement in both MSE and R^2 as the number of models increases from one to five, demonstrating the benefits of ensemble methods in reducing prediction errors. Beyond five models, marginal gains in performance become negligible, suggesting minimal returns with the inclusion of additional base models. Consequently, limiting the ensemble to five base models strikes an effective balance between predictive accuracy and computational efficiency.

S3.2. Scoring functions performance

A

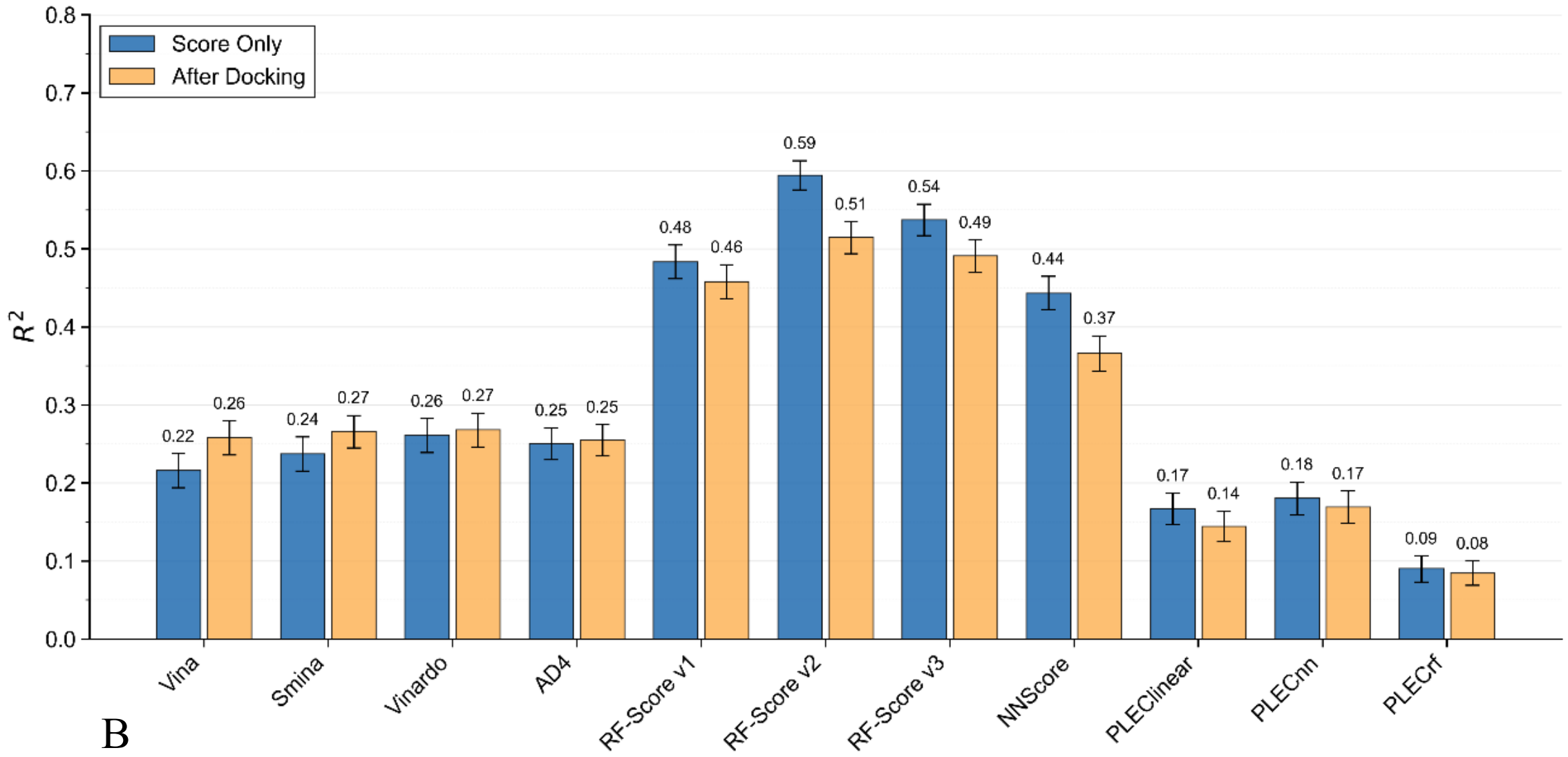

B

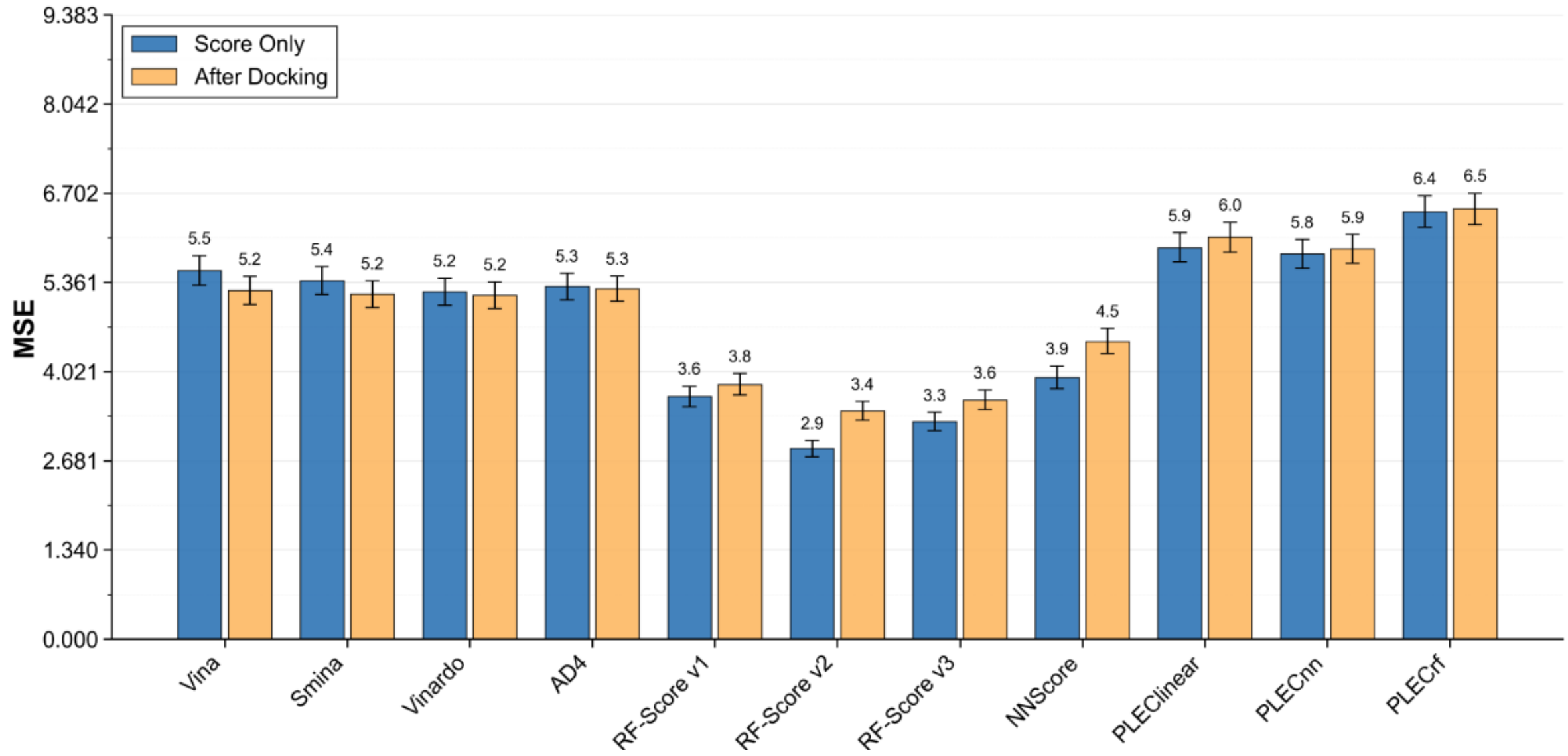

Figure S6 - R^2 (A) and MSE (B) of tested scoring functions evaluated on 4482 protein-ligand complexes from the PDBBind v2020 dataset. The blue bars correspond to performance on X-ray structures, while the yellow bar indicate results obtained after re-docking the ligands into protein binding pockets using AutoDock Vina, considering only the lowest-energy pose identified by Vina. Bars show the observed success rates, with error bars representing 95% percentile-bootstrap asymmetric confidence intervals calculated from 10000 resamples of the reference-variant pairs with replacement. The Vinardo scoring function exhibited the lowest MSE on X-ray structures but had a lower R^2 compared to several machine learning-based models. Traditional scoring functions such as Vina, Smina, and AD4 demonstrated comparable MSE and R^2 values. ML models generally improved predictive accuracy, except for PLEC Score functions, which showed poor performance with high MSE and low correlation. After docking, Vina and Vinardo, AD4 and Smina's performance in MSE showed slight improvements. Machine learning models maintained consistent correlation levels, with Rf-Score v2 achieving the highest R^2 value.

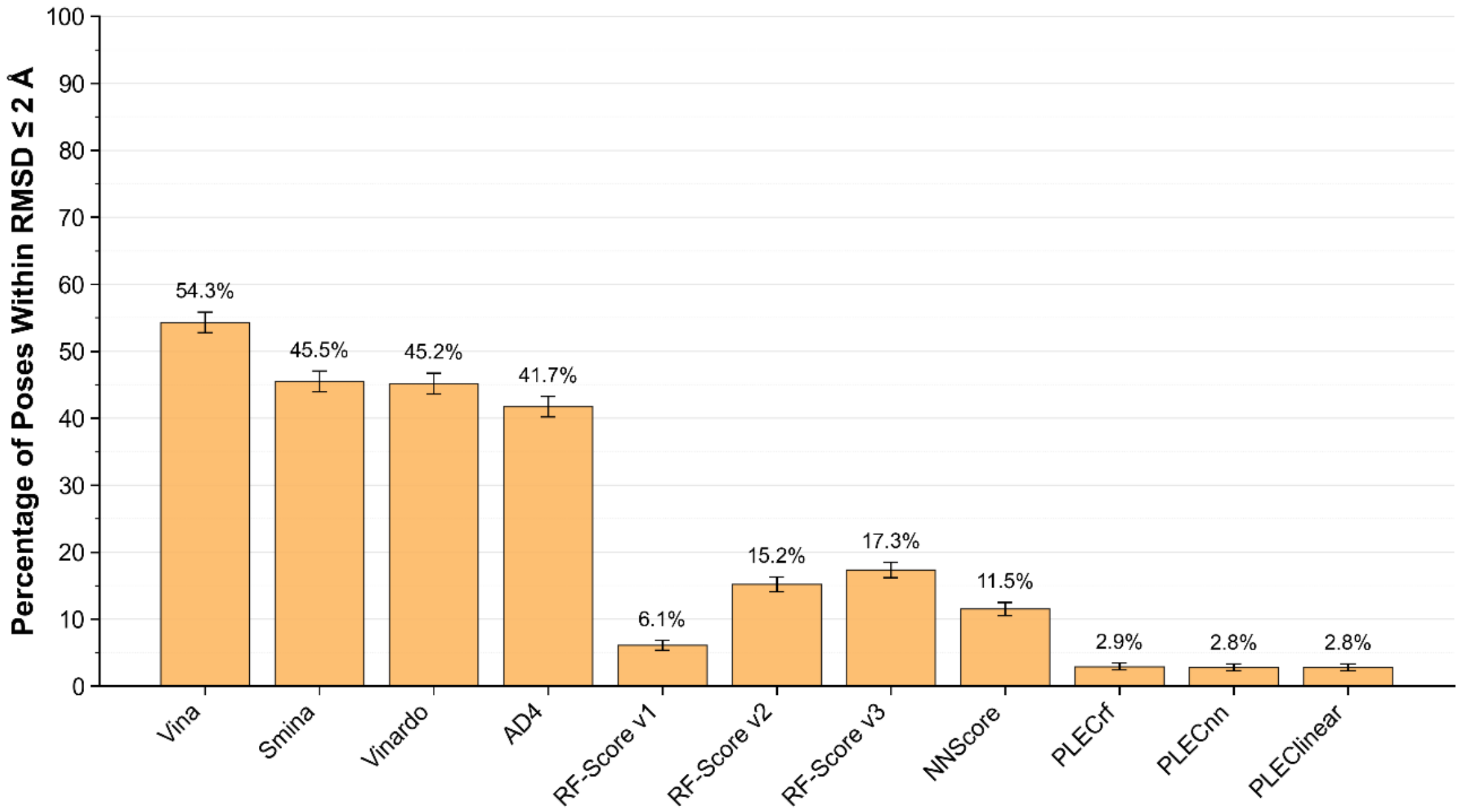

Figure S7 - Docking power evaluation of scoring functions on 4482 protein-ligand complexes. Docking power was measured as the percentage of predicted poses within 2 Å RMSD of the corresponding X-ray structure, among the lowest-energy poses identified by each scoring function. Bars show the observed success rates, with error bars representing 95% percentile-bootstrap asymmetric confidence intervals calculated from 10000 resamples of the reference-variant pairs with replacement. Among all tested methods, Vina achieved the highest docking power, outperforming all ML models. The generally lower performance of ML scoring functions in docking power may be attributed to their training primarily on experimented determined complexes rather than on docked poses, which limits their ability to accurately identify incorrect bound conformations.

A

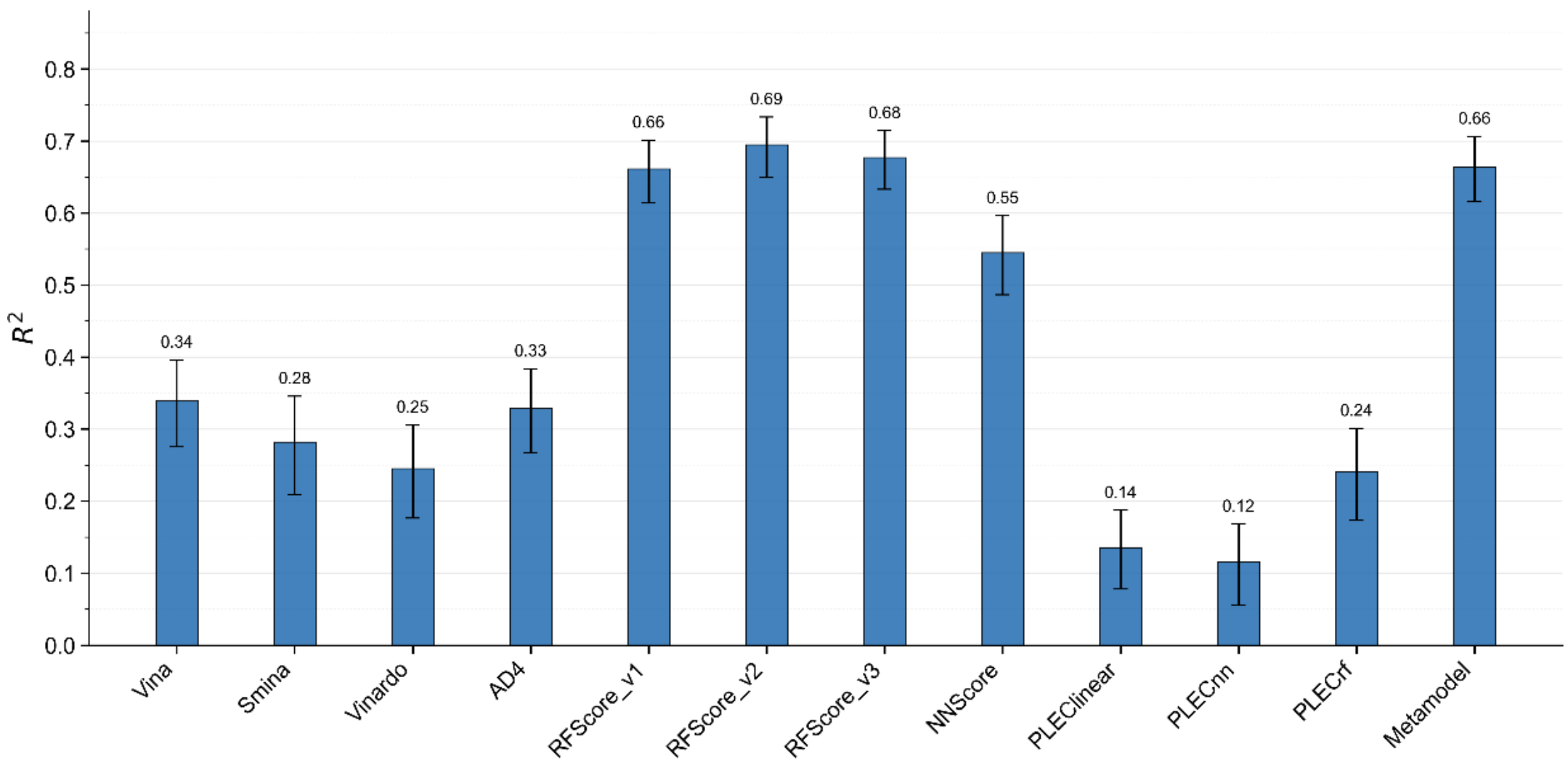

B

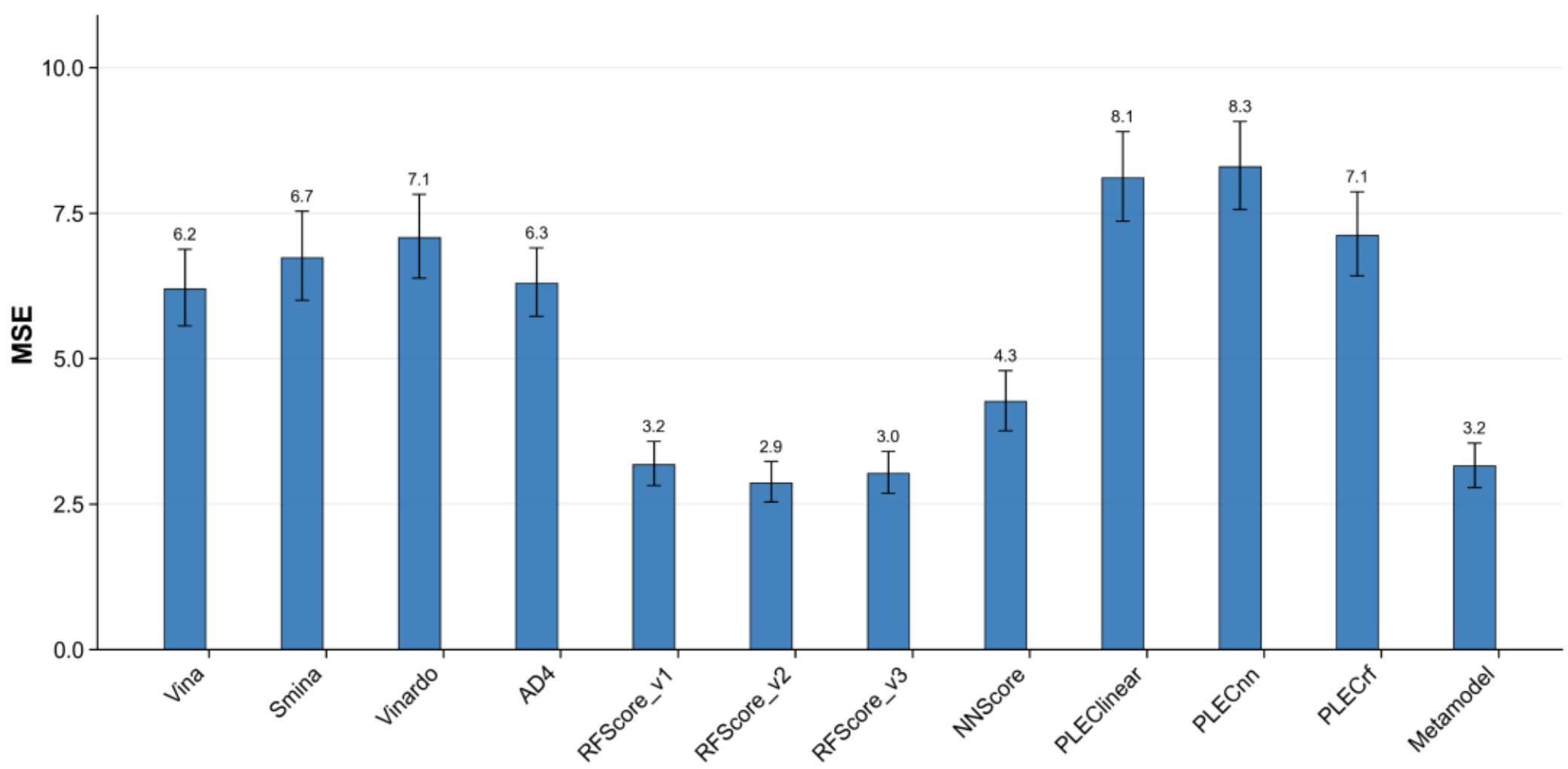

Figure S8 – Histogram comparison of R^2 (A) and MSE (B) between predicted and experimental binding affinities obtained through the platform's pipeline in the stacking validation set. Bars show the observed success rates, with error bars representing 95% percentile-bootstrap asymmetric confidence intervals calculated from 10000 resamples of the reference-variant pairs with replacement. The results are based on the lowest-energy pose identified by Vina. Among the scoring functions evaluated, the three versions of Rf-Score achieved the highest performance, both in R^2 and MSE, closely followed by the metamodel.

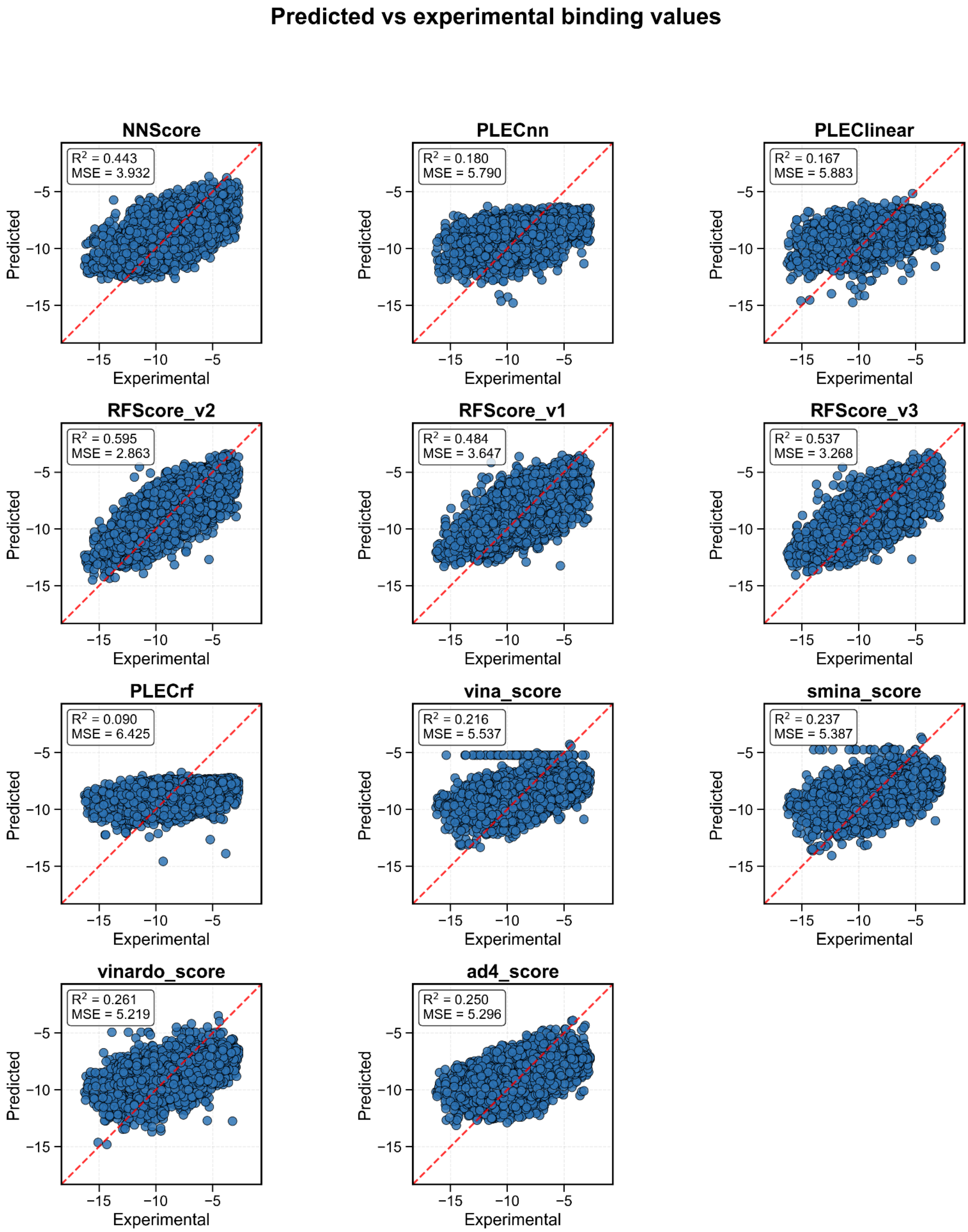
Predicted vs experimental binding values
NNScore
R² = 0.443
MSE = 3.932
PLECnn
R² = 0.180
MSE = 5.790
PLEClinear
R² = 0.167
MSE = 5.883
RFScore_v2
R² = 0.595
MSE = 2.863
RFScore_v1
R² = 0.484
MSE = 3.647
RFScore_v3
R² = 0.537
MSE = 3.268
PLECrf
R² = 0.090
MSE = 6.425
vina_score
R² = 0.216
MSE = 5.537
smina_score
R² = 0.237
MSE = 5.387
vinardo_score
R² = 0.261
MSE = 5.219
ad4_score
R² = 0.250
MSE = 5.296
Predicted
Experimental
−5
−10
−15

Figure S9 - Scatter plots comparing predicted binding affinities to experimental binding affinities. Predicted binding affinities when calculated using each scoring function on the crystallized pose (first phase). The ideal fit is represented in red. Structures from PDBBind v2020 database.

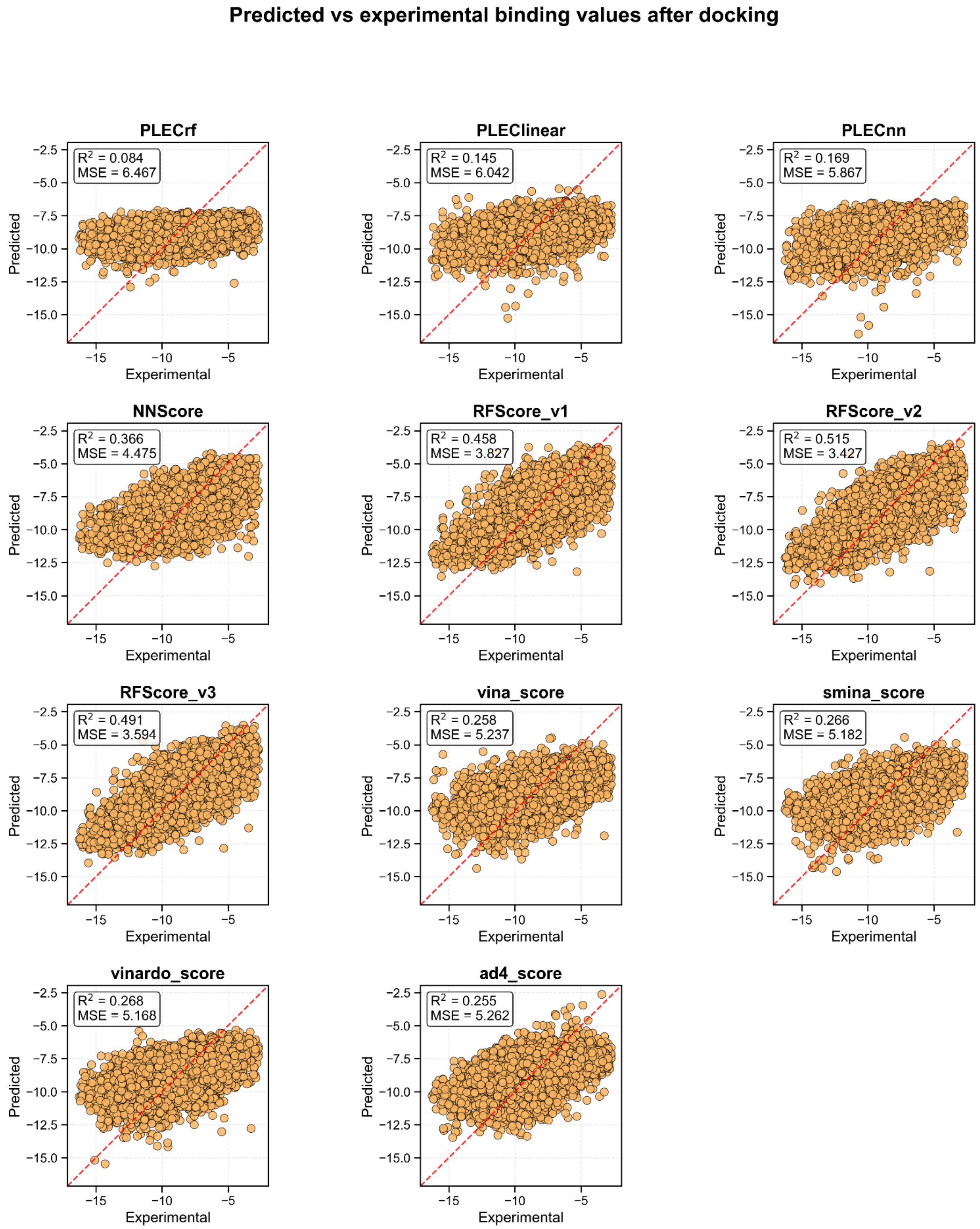

Figure S10 - Scatter plots comparing predicted binding affinities to experimental binding affinities. Predicted binding affinities after re-docking the ligand in the protein's binding pocket (second phase). The ideal fit is represented in red. Structures from PDBBind v2020 database.

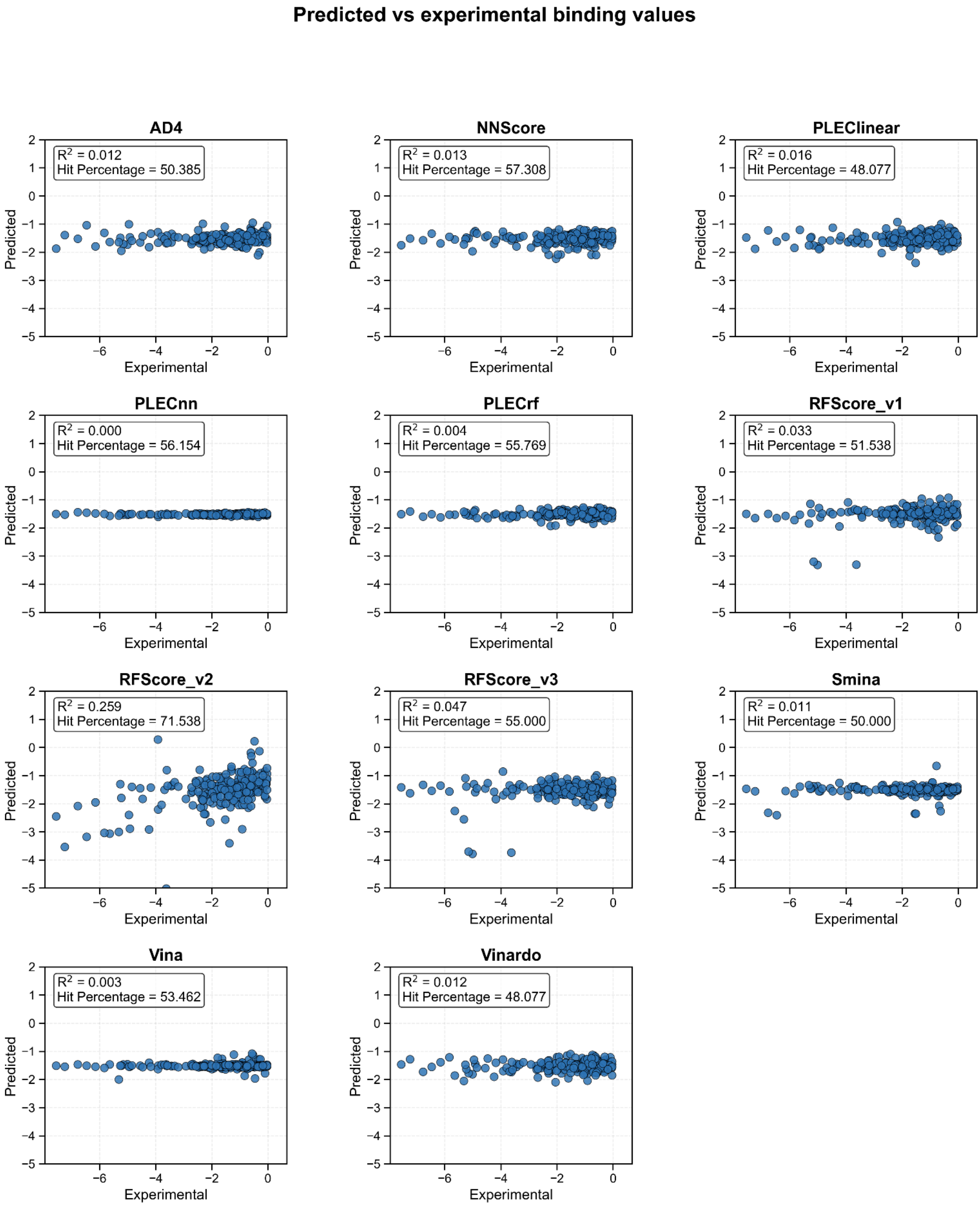

Figure S11 - Scatter plots comparing predicted ΔΔG to experimental ΔΔG. Predicted ΔΔG when calculated using each scoring function on the crystallized pose (first phase). Hits – Percentage of successes on predicting the mutation's effect on binding. Structures from PDBbind v2020 database.

Predicted vs experimental binding values after docking

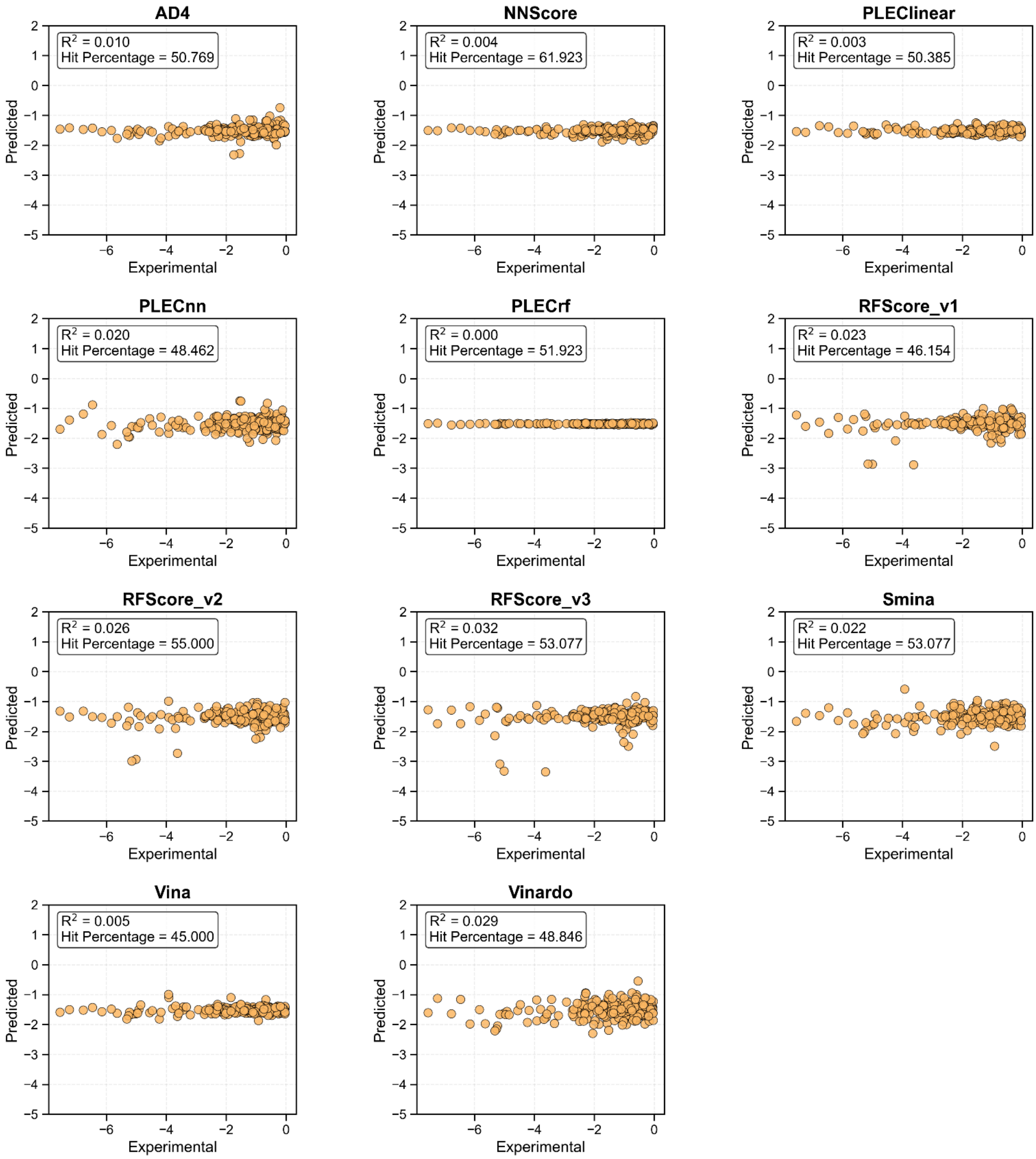

Figure S12 - Scatter plots comparing predicted ΔΔG to experimental ΔΔG. Predicted ΔΔG after re-docking the ligand in the protein's binding pocket (second phase). Hits – Percentage of successes on predicting the mutation's effect on binding. Structures from PDBbind v2020 database.

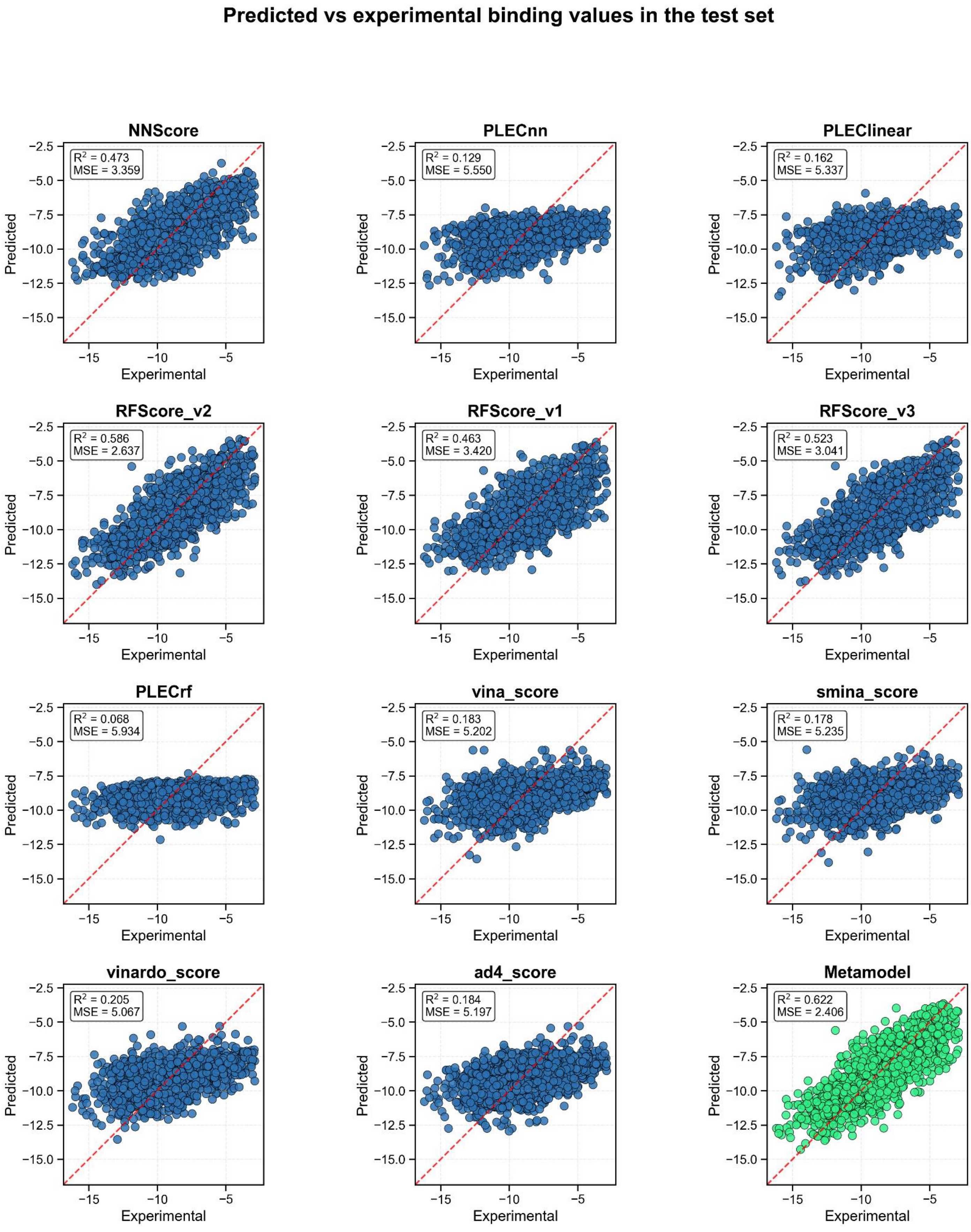

Figure S13 - Scatter plots comparing predicted binding affinities from the stacking test set to experimental binding affinities. Predicted binding affinities when calculated using each scoring function on the crystallized pose. The ideal fit is represented in red. Structures from PDBbind v2020 database.

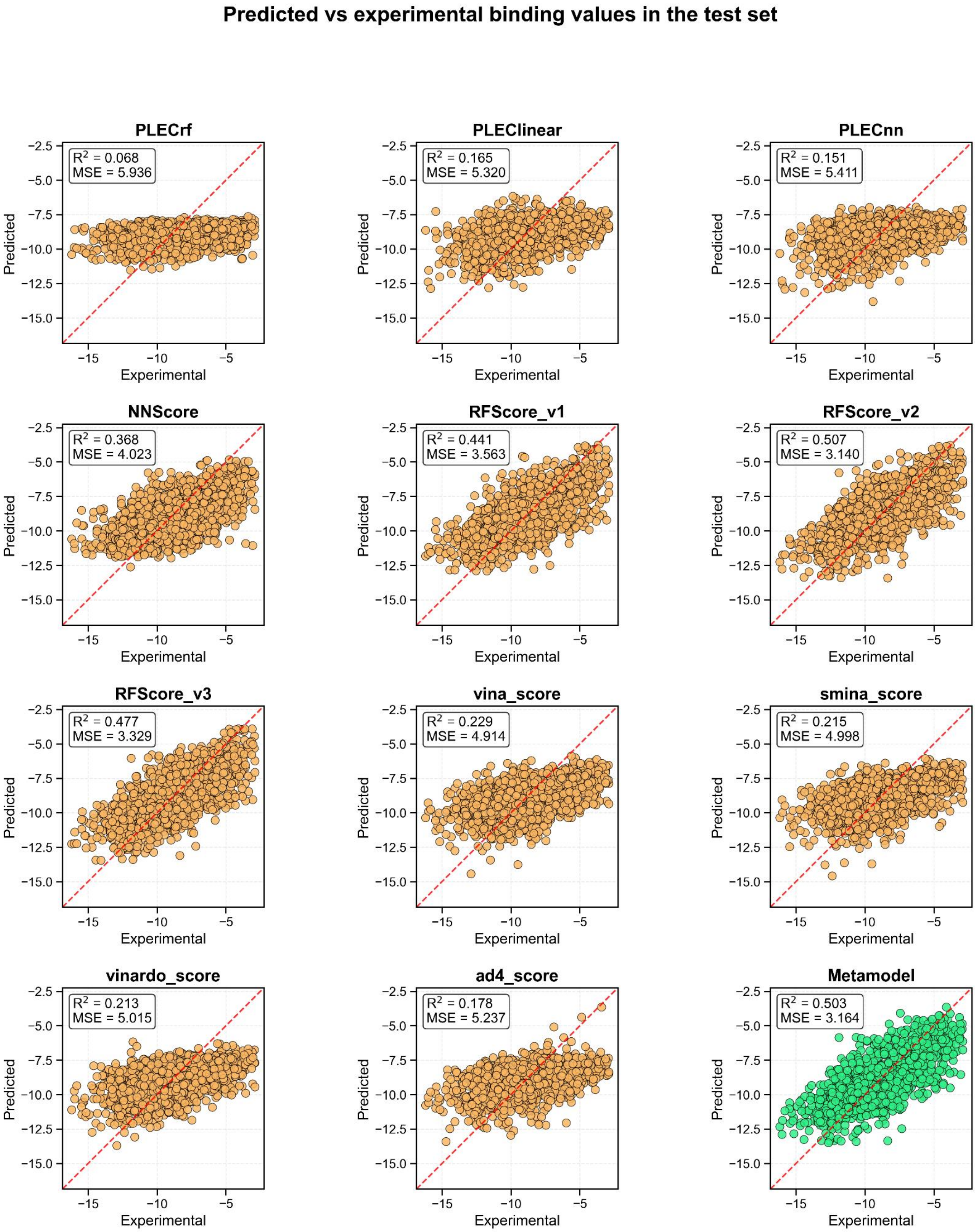

Figure S14 - Scatter plots comparing predicted binding affinities from the stacking test set to experimental binding affinities. Predicted binding affinities after re-docking the ligand in the protein's binding pocket. The ideal fit is represented in red. Structures from PDBbind v2020 database.

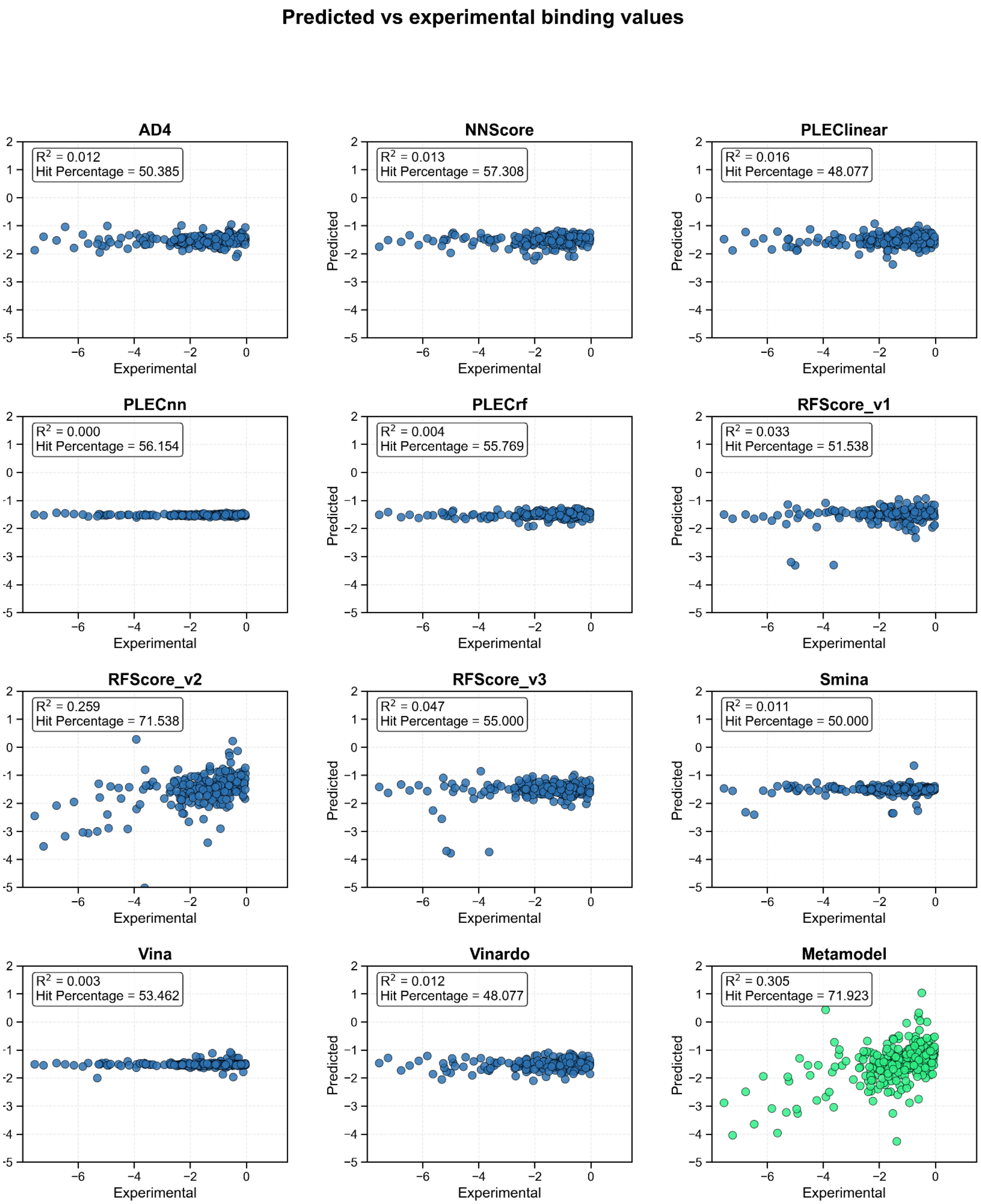

Figure S15 - Scatter plots comparing predicted ΔΔG to experimental ΔΔG for the stacking test set. Predicted ΔΔG when calculated using each scoring function on the crystallized pose (first phase). Hits – Percentage of successes on predicting the mutation's effect on binding. Structures from PDBbind v2020 database.

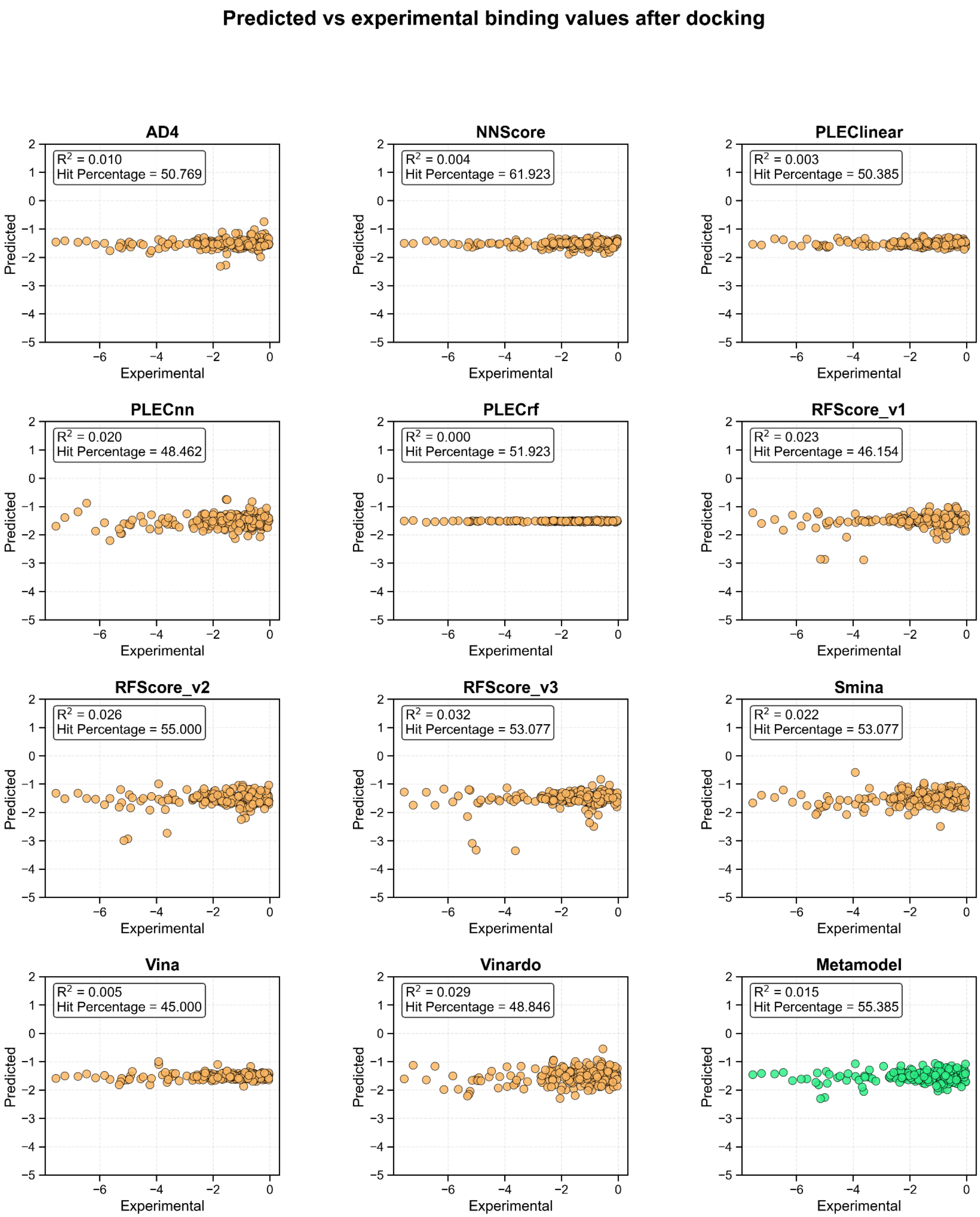

Figure S16 - Scatter plots comparing predicted ΔΔG to experimental ΔΔG for the stacking test set. Predicted ΔΔG after re-docking the ligand in the protein's binding pocket (second phase). Hits – Percentage of successes on predicting the mutation's effect on binding. Structures from PDBbind v2020 database.

Predicted vs experimental binding values in the platform pipeline

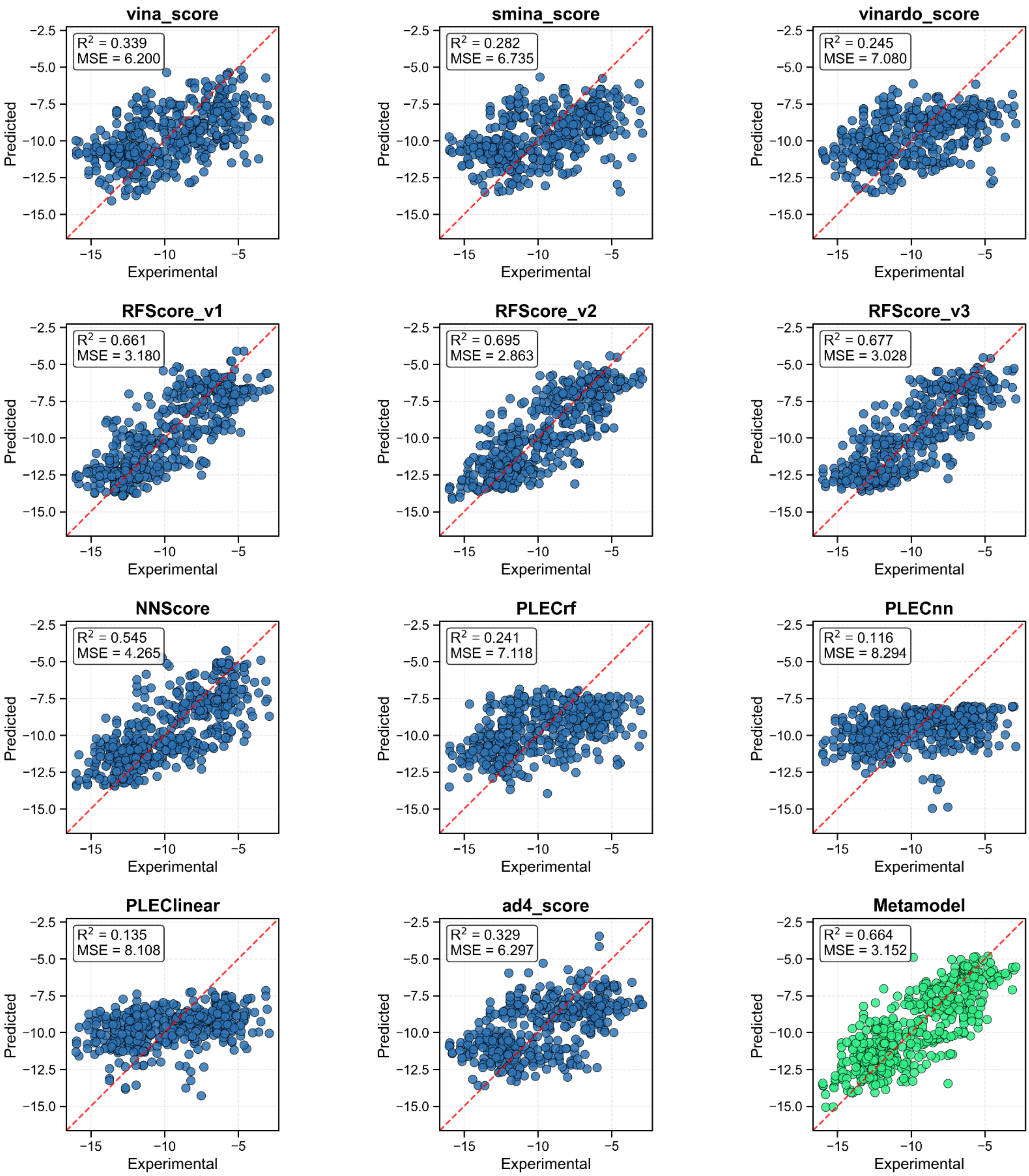

Figure S17 - Scatter plots comparing predicted binding affinities from the platform's results to experimental binding affinities for the stacking test set. The ideal fit is represented in red. Structures from PDBBind v2020 database.

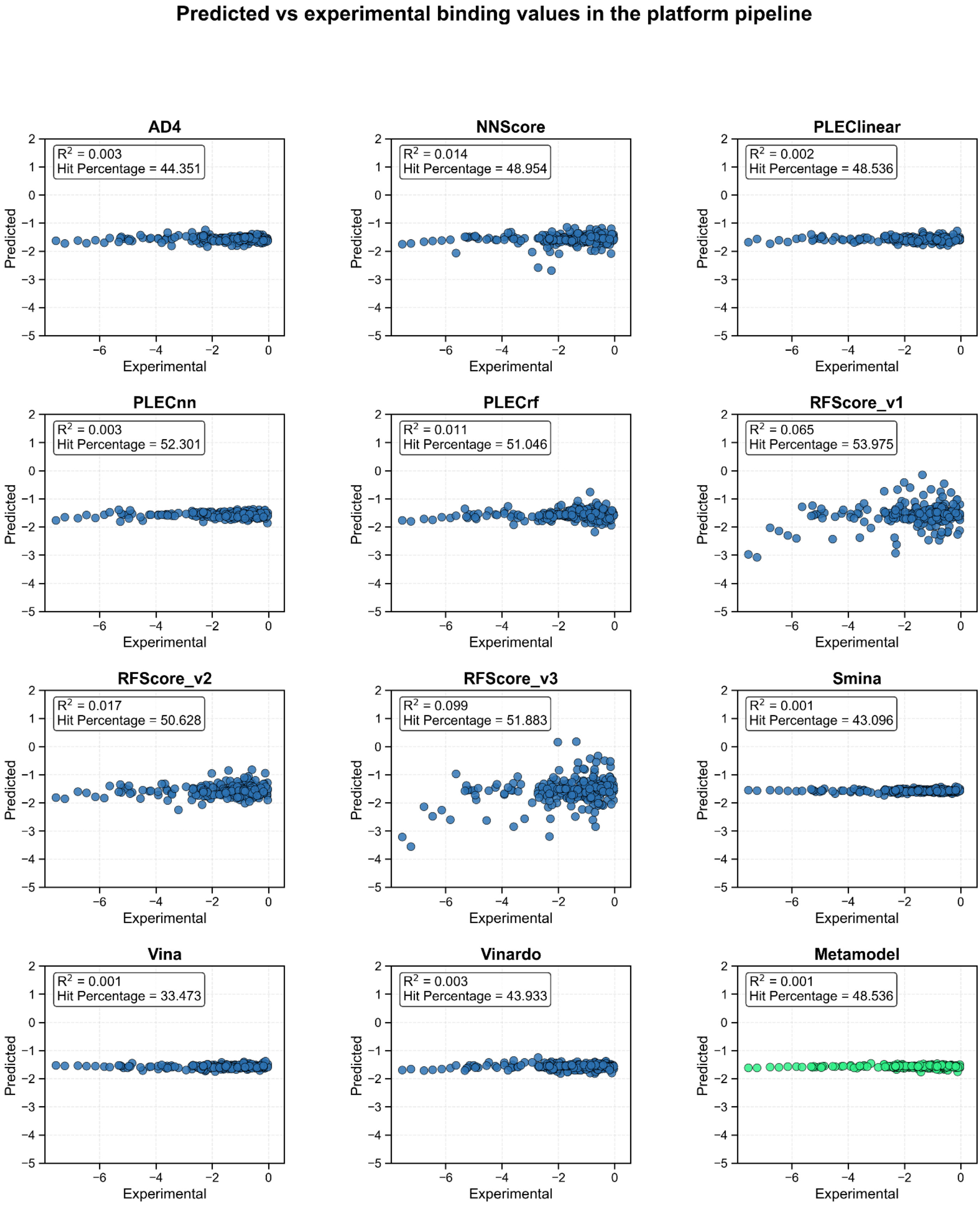

Figure S18 - Scatter plots comparing predicted ΔΔG from the platform's results to experimental ΔΔG for the stacking test set. Predicted ΔΔG when considering the best pose found by Vina. Hits – Percentage of successes on predicting the mutation's effect on binding. Structures from PDBbind v2020 database.

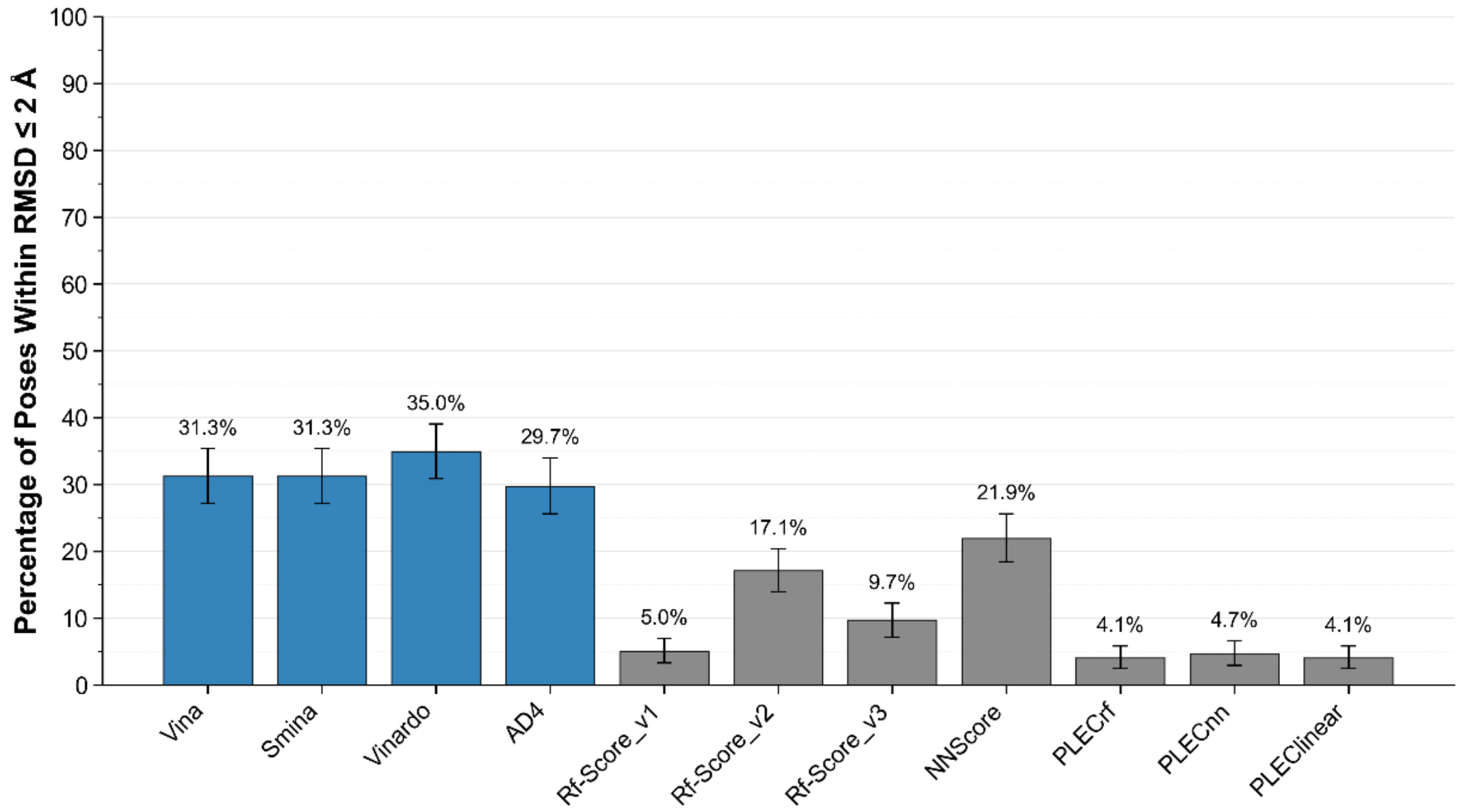

Figure S19 - Docking power prediction of the results obtained by the platform. Docking ability measured as a percentage of poses, for the 260 pairs of protein-ligand complexes, within a 2 Å RMSD from the crystalized one which the scoring function predicted as best pose. Bars show the observed success rates, with error bars representing 95% percentile-bootstrap asymmetric confidence intervals calculated from 10000 resamples of the reference-variant pairs with replacement. Classical scoring functions are represented in blue and machine learning-based scoring functions in gray.

REFERENCES

(1) Du, J.; Shao, Z.; Zhao, H. Engineering Microbial Factories for Synthesis of Value-Added Products. *J. Ind. Microbiol. Biotechnol.* **2011**, *38* (8), 873–890. https://doi.org/10.1007/s10295-011-0970-3.

(2) Geraldi, A.; Khairunnisa, F.; Farah, N.; Bui, L. M.; Rahman, Z. Synthetic Scaffold Systems for Increasing the Efficiency of Metabolic Pathways in Microorganisms. *Biology (Basel).* **2021**, *10* (3), 216. https://doi.org/10.3390/biology10030216.

(3) Pleiss, J. Protein Design in Metabolic Engineering and Synthetic Biology. *Curr. Opin. Biotechnol.* **2011**, *22* (5), 611–617. https://doi.org/10.1016/j.copbio.2011.03.004.

(4) Wang, L.; Gu, J.; Zhao, W.; Wang, M.; Ng, K. R.; Lyu, X.; Yang, R. Reshaping the Binding Pocket of Cellobiose 2-Epimerase for Improved Substrate Affinity and Isomerization Activity for Enabling Green Synthesis of Lactulose. *J. Agric. Food Chem.* **2022**, *70* (50), 15879–15893. https://doi.org/10.1021/acs.jafc.2c06980.

(5) Bloom, J. D.; Meyer, M. M.; Meinhold, P.; Otey, C. R.; MacMillan, D.; Arnold, F. H. Evolving Strategies for Enzyme Engineering. *Curr. Opin. Struct. Biol.* **2005**, *15* (4), 447–452. https://doi.org/10.1016/j.sbi.2005.06.004.

(6) Marcheschi, R. J.; Li, H.; Zhang, K.; Noey, E. L.; Kim, S.; Chaubey, A.; Houk, K. N.; Liao, J. C. A Synthetic Recursive "+1" Pathway for Carbon Chain Elongation. *ACS Chem. Biol.* **2012**, *7* (4), 689–697. https://doi.org/10.1021/cb200313e.

(7) Ranaghan, K. E.; Mulholland, A. J. Investigations of Enzyme-Catalysed Reactions with Combined Quantum Mechanics/Molecular Mechanics (QM/MM) Methods. *Int. Rev. Phys. Chem.* **2010**, *29* (1), 65–133. https://doi.org/10.1080/01442350903495417.

(8) Kiss, G.; Çelebi-Ölçüm, N.; Moretti, R.; Baker, D.; Houk, K. N. Computational Enzyme Design. *Angew. Chemie - Int. Ed.* **2013**, *52* (22), 5700–5725. https://doi.org/10.1002/anie.201204077.

(9) Morris, G. M.; Lim-Wilby, M. Molecular Docking. *Methods Mol. Biol.* **2008**, *443*, 365–382. https://doi.org/10.1007/978-1-59745-177-2_19.

(10) Sirin, S.; Kumar, R.; Martinez, C.; Karmilowicz, M. J.; Ghosh, P.; Abramov, Y. A.; Martin, V.; Sherman, W. A Computational Approach to Enzyme Design: Predicting W-Aminotransferase Catalytic Activity Using Docking and MM-GBSA Scoring. *J. Chem. Inf. Model.* **2014**, *54* (8), 2334–2346. https://doi.org/10.1021/ci5002185.

(11) Trott, O.; Olson, A. J. AutoDock Vina: Improving the Speed and Accuracy of Docking with a New Scoring Function, Efficient Optimization, and Multithreading. *J. Comput. Chem.* **2010**, *31* (2), 455–461. https://doi.org/10.1002/jcc.21334.

(12) Chu, W. T.; Wang, J. Energy Landscape Topography Reveals the Underlying Link between Binding Specificity and Activity of Enzymes. *Sci. Rep.* **2016**, *6*, 27808. https://doi.org/10.1038/srep27808.

(13) Xia, W.; Bai, Y.; Shi, P. Improving the Substrate Affinity and Catalytic Efficiency of β-Glucosidase Bgl3A from Talaromyces Leycettanus JCM12802 by Rational Design. *Biomolecules* **2021**, *11* (12), 1882. https://doi.org/10.3390/biom11121882.

(14) Su, D.; Kosciuk, T.; Yang, M.; Price, I. R.; Lin, H. Binding Affinity Determines Substrate Specificity and Enables Discovery of Substrates for N-Myristoyltransferases. *ACS Catal.* **2021**, *11* (24), 14877–14883. https://doi.org/10.1021/acscatal.1c03330.

(15) Abramson, J.; Adler, J.; Dunger, J.; Evans, R.; Green, T.; Pritzel, A.; Ronneberger, O.; Willmore, L.; Ballard, A. J.; Bambrick, J.; Bodenstein, S. W.; Evans, D. A.; Hung, C. C.; O'Neill, M.; Reiman, D.; Tunyasuvunakool, K.; Wu, Z.; Žemgulytė, A.; Arvaniti, E.; Beattie, C.; Bertolli, O.; Bridgland, A.; Cherepanov, A.; Congreve, M.; Cowen-Rivers, A. I.; Cowie, A.; Figurnov, M.; Fuchs, F. B.; Gladman, H.; Jain, R.; Khan, Y. A.; Low, C. M. R.; Perlin, K.; Potapenko, A.; Savy, P.; Singh, S.; Stecula, A.; Thillaisundaram, A.; Tong, C.; Yakneen, S.; Zhong, E. D.; Zielinski, M.; Žídek, A.; Bapst, V.; Kohli, P.; Jaderberg, M.; Hassabis, D.; Jumper, J. M. Accurate Structure Prediction of Biomolecular Interactions with AlphaFold 3. *Nature* **2024**, *630* (8016), 493–500. https://doi.org/10.1038/s41586-024-07487-w.

(16) Šali, A.; Blundell, T. L. Comparative Protein Modelling by Satisfaction of Spatial Restraints. *J. Mol. Biol.* **1993**, *234* (3), 779–815. https://doi.org/10.1006/jmbi.1993.1626.

(17) Koes, D. R.; Baumgartner, M. P.; Camacho, C. J. Lessons Learned in Empirical Scoring with Smina from the CSAR 2011 Benchmarking Exercise. *J. Chem. Inf. Model.* **2013**, *53* (8), 1893–1904. https://doi.org/10.1021/ci300604z.

(18) Dalcin, L.; Fang, Y. L. L. Mpi4py: Status Update after 12 Years of Development. *Comput. Sci. Eng.* **2021**, *23* (4), 47–54. https://doi.org/10.1109/MCSE.2021.3083216.

(19) Olechnovič, K.; Venclovas, Č. VoroMQA: Assessment of Protein Structure Quality Using Interatomic Contact Areas. *Proteins Struct. Funct. Bioinforma.* **2017**, *85* (6), 1131–1145. https://doi.org/10.1002/prot.25278.

(20) Shen, M.; Sali, A. Statistical Potential for Assessment and Prediction of Protein Structures. *Protein Sci.* **2006**, *15* (11), 2507–2524. https://doi.org/10.1110/ps.062416606.

(21) Morris, G. M.; Ruth, H.; Lindstrom, W.; Sanner, M. F.; Belew, R. K.; Goodsell, D. S.; Olson, A. J. Software News and Updates AutoDock4 and AutoDockTools4: Automated Docking with Selective Receptor Flexibility. *J. Comput. Chem.* **2009**, *30* (16), 2785–2791. https://doi.org/10.1002/jcc.21256.

(22) Liu, Z.; Su, M.; Han, L.; Liu, J.; Yang, Q.; Li, Y.; Wang, R. Forging the Basis for Developing Protein-Ligand Interaction Scoring Functions. *Acc. Chem. Res.* **2017**, *50* (2), 302–309. https://doi.org/10.1021/acs.accounts.6b00491.

(23) Wang, R.; Fang, X.; Lu, Y.; Wang, S. The PDBbind Database: Collection of Binding Affinities for Protein-Ligand Complexes with Known Three-Dimensional Structures. *J. Med. Chem.* **2004**, *47* (12), 2977–2980. https://doi.org/10.1021/jm030580l.

(24) Wang, R.; Fang, X.; Lu, Y.; Yang, C. Y.; Wang, S. The PDBbind Database: Methodologies and Updates. *J. Med. Chem.* **2005**, *48* (12), 4111–4119. https://doi.org/10.1021/jm048957q.

(25) Quiroga, R.; Villarreal, M. A. Vinardo: A Scoring Function Based on Autodock Vina Improves Scoring, Docking, and Virtual Screening. *PLoS One* **2016**, *11* (5), e0155183. https://doi.org/10.1371/journal.pone.0155183.

(26) Hanko, E. K. R.; Correia, J.; Souza, C. S.; Green, A.; Chromy, J.; Stoney, R.; Yan, C.; Takano, E.; Lousa, D.; Soares, C. M.; Breitling, R. Microbial Production of the Plant Flavanone Hesperetin from Caffeic Acid. *BMC Res. Notes* **2023**, *16* (1), 343. https://doi.org/10.1186/s13104-023-06620-8.

(27) Ferreira, S.; Balola, A.; Sveshnikova, A.; Hatzimanikatis, V.; Vilaça, P.; Maia, P.; Carreira, R.; Stoney, R.; Carbonell, P.; Souza, C. S.; Correia, J.; Lousa, D.; Soares, C. M.; Rocha, I. Computer-Aided Design and Implementation of Efficient Biosynthetic Pathways to Produce High Added-Value Products Derived from Tyrosine in Escherichia Coli. *Front. Bioeng. Biotechnol.* **2024**, *12* (June), 1–20. https://doi.org/10.3389/fbioe.2024.1360740.

(28) Durrant, J. D.; McCammon, J. A. NNScore: A Neural-Network-Based Scoring Function for the Characterization of Protein-Ligand Complexes. *J. Chem. Inf. Model.* **2010**, *50* (10), 1865–1871. https://doi.org/10.1021/ci100244v.

(29) Pereira, J. C.; Caffarena, E. R.; Dos Santos, C. N. Boosting Docking-Based Virtual Screening with Deep Learning. *J. Chem. Inf. Model.* **2016**, *56* (12), 2495–2506. https://doi.org/10.1021/acs.jcim.6b00355.

(30) Ballester, P. J.; Mitchell, J. B. O. A Machine Learning Approach to Predicting Protein-Ligand Binding Affinity with Applications to Molecular Docking. *Bioinformatics* **2010**, *26* (9), 1169–1175. https://doi.org/10.1093/bioinformatics/btq112.

(31) Li, H.; Leung, K. S.; Wong, M. H.; Ballester, P. J. Improving Autodock Vina Using Random Forest: The Growing Accuracy of Binding Affinity Prediction by the Effective Exploitation of Larger Data Sets. *Mol. Inform.* **2015**, *34* (2–3), 115–126. https://doi.org/10.1002/minf.201400132.

(32) Ballester, P. J.; Schreyer, A.; Blundell, T. L. Does a More Precise Chemical Description of Protein-Ligand Complexes Lead to More Accurate Prediction of Binding Affinity? *J. Chem. Inf. Model.* **2014**, *54* (3), 944–955. https://doi.org/10.1021/ci500091r.

(33) Durrant, J. D.; McCammon, J. A. NNScore 2.0: A Neural-Network Receptor-Ligand Scoring Function. *J. Chem. Inf. Model.* **2011**, *51* (11), 2897–2903. https://doi.org/10.1021/ci2003889.

(34) Wójcikowski, M.; Kukiełka, M.; Stepniewska-Dziubinska, M. M.; Siedlecki, P. Development of a Protein-Ligand Extended Connectivity (PLEC) Fingerprint and Its Application for Binding Affinity Predictions. *Bioinformatics* **2019**, *35* (8), 1334–1341. https://doi.org/10.1093/bioinformatics/bty757.

(35) Mohamed Abdul Cader, J.; Newton, M. A. H.; Rahman, J.; Mohamed Abdul Cader, A. J.; Sattar, A. Ensembling Methods for Protein-Ligand Binding Affinity Prediction. *Sci. Rep.* **2024**, *14* (1), 1–19. https://doi.org/10.1038/s41598-024-72784-3.

(36) Lee, H. J.; Emani, P. S.; Gerstein, M. B. Improved Prediction of Ligand-Protein Binding Affinities by Meta-Modeling. *J. Chem. Inf. Model.* **2024**, *64* (23), 8684–8704. https://doi.org/10.1021/acs.jcim.4c01116.
